# Global, site-specific analysis of neuronal protein S-acylation

**DOI:** 10.1038/s41598-017-04580-1

**Published:** 2017-07-05

**Authors:** Mark O. Collins, Keith T. Woodley, Jyoti S. Choudhary

**Affiliations:** 1 Wellcome Trust Sanger Institute, Hinxton, Cambridge CB10 1SA UK; 2 Department of Biomedical Science & Centre for Membrane Interactions and Dynamics (CMIAD), Firth Court, Western Bank, University of Sheffield, Sheffield, S10 2TN UK

## Abstract

Protein S-acylation (palmitoylation) is a reversible lipid modification that is an important regulator of dynamic membrane-protein interactions. Proteomic approaches have uncovered many putative palmitoylated proteins however, methods for comprehensive palmitoylation site characterization are lacking. We demonstrate a quantitative site-specific-Acyl-Biotin-Exchange (ssABE) method that allowed the identification of 906 putative palmitoylation sites on 641 proteins from mouse forebrain. 62% of sites map to known palmitoylated proteins and 102 individual palmitoylation sites are known from the literature. 54% of palmitoylation sites map to synaptic proteins including many GPCRs, receptors/ion channels and peripheral membrane proteins. Phosphorylation sites were also identified on a subset of peptides that were palmitoylated, demonstrating for the first time co-identification of these modifications by mass spectrometry. Palmitoylation sites were identified on over half of the family of palmitoyl-acyltransferases (PATs) that mediate protein palmitoylation, including active site thioester-linked palmitoyl intermediates. Distinct palmitoylation motifs and site topology were identified for integral membrane and soluble proteins, indicating potential differences in associated PAT specificity and palmitoylation function. ssABE allows the global identification of palmitoylation sites as well as measurement of the active site modification state of PATs, enabling palmitoylation to be studied at a systems level.

## Introduction

Protein S-palmitoylation, also known as S-acylation, is a thioester linkage of a lipid to cysteine residues in proteins, with the 16-carbon fatty acid palmitate being the most common type. Palmitoylation is the only known reversible lipid modification of proteins and can therefore act as a regulatory post-translational modification. Proteins can be palmitoylated by a family of 23 mammalian palmitoyl-acyltransferases (PATs) that contain DHHC domains, the catalytic domain of these enzymes containing a conserved “DHHC” sequence motif with the cysteine residue carrying a thioester linked palmitoyl intermediate that is transferred to substrate proteins^1^. Proteins are depalmitoylated by two cytosolic acyl-protein thioesterases^2, 3^ (APT1 and APT2), PPT1^4^ and a recently discovered family of ABHD17 proteins^5^.

Palmitoylation increases hydrophobicity at specific points in protein structures that allow tethering/interaction of proteins to membranes, a process that can regulate protein trafficking, compartmentalization and cell surface expression. Classic examples of soluble proteins that associate with membranes through palmitoylation are Ras^6^ and PSD-95^7^. Integral membrane proteins are also widely regulated by palmitoylation; controlling for example trafficking of receptors to the plasma membrane as well as their internalization when no longer required at the cell surface. Palmitoylation can also regulate the topology/structure of integral membrane proteins by for example tethering cytoplasmic tails to the membrane and there by masking or indeed creating sites for interacting proteins to bind as well as regulating the accessibility of protein domains to enzymes that mediate other regulatory PTMs^8^.

The characterization of palmitoylated proteins on a proteome scale has been accelerated by the development of purification methods that selectively replace palmitoyl groups with affinity purification tags^9^ or metabolic labelling^10^ with palmitic acid analogues that are selectively captured. Acyl-biotin exchange (ABE)^11^ was adapted by Davis and co-workers^12^ for proteomic applications and involves blocking all free cysteines in proteins, subsequent release of palmitoyl thioester bonds using hydroxylamine at neutral pH^11^ and biotinylation of newly exposed cysteine thiol groups. Labelled proteins can then be purified using streptavidin resin and identified using LC-MS/MS analysis. The second approach developed by Hang and co-workers^13^ and subsequently by Martin and Cravatt^10^ involves metabolic labelling of cells with bio-orthogonal probes of palmitoylation. The palmitic acid analogue 17-ODYA is incorporated into proteins at sites of palmitoylation and click-chemistry is used to specifically label 17-OYDA modified proteins with biotin which can be used to purify proteins palmitoylated during the period of metabolic labelling for characterization by LC-MS/MS analysis. Both ABE and click chemistry based approaches can be combined with quantitative proteomic workflows to enable relative quantification of palmitoylated proteins compared to appropriate controls as well as relative levels of palmitoylated proteins in perturbation experiments^14^.

The combination of these enrichment methods with mass spectrometry has enabled the identification of many palmitoylated proteins from a range of cells, tissues and species^9, 10, 14–16^. Global palmitoyl-proteome analysis has shown that this modification is relatively common and occurs on proteins involved in a range of biological processes. However, such approaches lack the resolution to identify exact sites of palmitoylation, the number of palmitoylation sites on a given protein or the potential to measure differentially regulated palmitoylation sites which is an extra level of complexity that is emerging in the field^17, 18^. Recently, attempts have been made to develop proteomic approaches that generate site specific data. Yang and co-workers adapted the ABE method by performing free cysteine blocking, hydroxylamine treatment and biotin labelling steps in a similar manner as the standard method but next performed tryptic digestion and purified biotinylated peptides using streptavidin resin^19^. Eluted peptides were analyzed by LC-MS/MS analysis and the relative enrichment of peptides in hydroxylamine treated compared to control samples was assessed by spectral counting. 25 known and 143 candidate palmitoylation sites were reported, however﻿, the confidence in palmitoylation site identification was uncertain due to the type of quantification employed. Recently, an alternative approach involving resin assisted capture (acyl-RAC) was developed by Forrester and co-workers to identify sites of S-acylation^20^. In this strategy, the biotin/avidin purification step is replaced by capture of previously palmitoylated cysteine thiols on a thiol-reactive resin and protein digestion is performed on-resin. This approach allowed the identification of 84 peptides containing putative sites of S-acylation but quantification of enrichment of these peptides in hydroxylamine treated compared to control samples was not reported.

In order to build upon and extend the scope of these approaches, we have developed a modified peptide-centric version of acyl-biotin exchange designated ssABE (site-specific Acyl-Biotin Exchange), in which most of the protocol is performed in a single reaction chamber in a fraction of the time as the traditional acyl-biotin exchange method, allowing it to be used as a site-specific palmitoylation assay.

## Results and Discussion

### ssABE-a strategy for mapping palmitoylation sites in endogenous proteins

**Figure 1:**
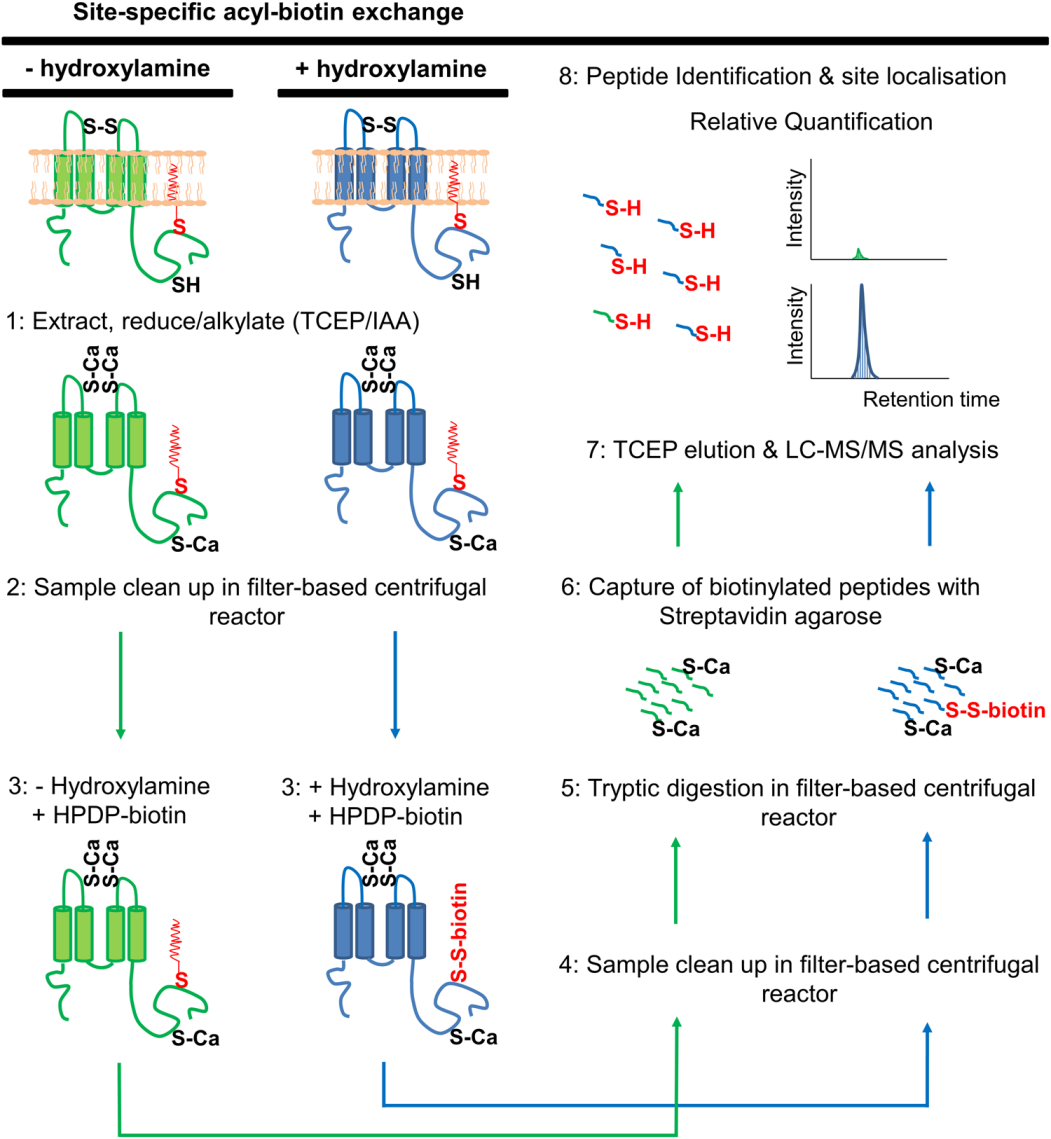
Schematic representation of the ssABE workflow. Protein samples are (1) reduced and alkylated and transferred to a filter-based centrifugal unit in which the following steps are performed; (2) rapid removal of reagents, (3) release of palmitoyl groups from proteins by hydroxylamine treatment at neutral pH and concomitant biotinylation of these newly “free” cysteine residues with a TCEP-cleavable biotin (HPDP-biotin) (4) rapid removal of reagents, followed by (5) digestion of proteins into peptides. (6) Biotinylated peptides are captured on Streptavidin agarose beads, washed and eluted (using TCEP) as free cysteine-containing peptides and (7) identified and quantified by LC-MS/MS analysis. Quantitative enrichment of peptides in +/− hydroxylamine treated samples allows identification of previously palmitoylated peptides. Palmitoylation sites are localized in peptide sequences by the presence of free (non-alkylated) cysteine residues.

The standard ABE method involves eight chloroform-methanol precipitation steps to remove reduction/alkylation reagents and biotin^9^ which make it a lengthy procedure and also results in significant protein loss. Centrifugal molecular weight-based cut-off concentrators are now commonly used for sample clean-up and proteolytic digestion^21^ and more recently for enrichment of post-translational modifications^22^. We extracted protein from adult mouse forebrain samples in a high SDS-containing buffer in order to maximize recovery of integral membrane proteins, which also contains TCEP and iodoacetamide for concurrent disulfide bond reduction and cysteine alkylation. Reduced and alkylated protein samples are then buffer exchanged using 8 M urea in a centrifugal concentrator. Critically, sample clean-up, hydroxylamine treatment, biotin labelling of previously palmitoylated cysteines, biotin removal and tryptic digestion steps are all performed in the same chamber of the concentrator to minimize sample loss and to greatly increase the speed in which the method can be performed (Fig. 1). After tryptic digestion, samples are removed from the chamber and incubated with streptavidin resin. Stringent washes are performed to remove non-specifically bound peptides and biotinylated peptides are selectively released by virtue of TCEP cleavable HPDP-biotin. Eluted peptides are acidified and analyzed by LC-MS/MS and unmodified cysteine resides in peptides quantitatively enriched in hydroxylamine treated compared to control samples are indicative of palmitoylation sites.

**Figure 2:**
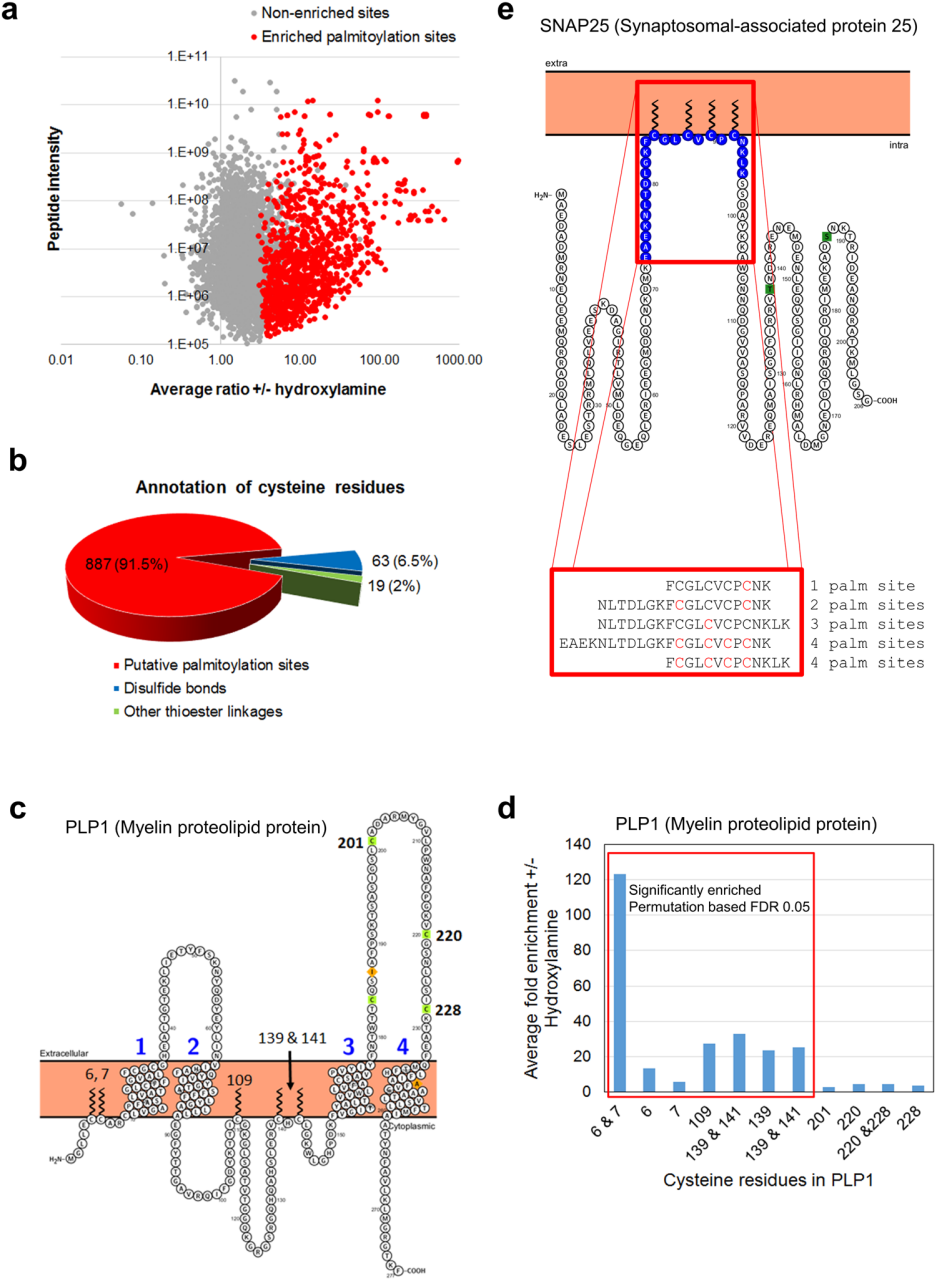
Identification of palmitoylation sites by analysis of peptide enrichment in +/− hydroxylamine treated samples. (**a**) Six biological replicates (2 technical replicates for each) each for +/− hydroxylamine treatments were used for label free quantification. Data points in red are significantly enriched (0.05 permutation-based FDR) in the hydroxylamine treated compared to the control set. (**b**) Putative palmitoylation sites were annotated with data from UniProt to find potential false positives. 2% of sites are annotated as being involved in other types of thioester linkages and 6.5% are potentially involved in predicted disulfide bonds but sites for disulfide bonds and palmitoylation are not mutually exclusive. (**c**) PLP1 contains multiple sites of palmitoylation and disulfide bonds. 5 palmitoylation sites were significantly enriched by ssABE whilst peptides bearing cysteine resides involved in the formation of disulfide bonds were not enriched upon treatment with hydroxylamine (**d**). (**e**) High coverage of multi-palmitoylated proteins such as the t-snare SNAP25 is obtained using ssABE. All 4 known palmitoylation sites on SNAP25 were identified and multiple palmitoylation states were independently observed (on Cys 85, 88, 90 & 92) on a number of different peptides.

The fully optimized ssABE method was used to label and purify palmitoylated peptides from six biological replicates each of hydroxylamine treated and control mouse forebrain lysates. Peptides from each purification were analyzed in duplicate by LC-MS/MS. Data was analyzed with MaxQuant^23^ and quantitation performed using peptide intensities with match between runs enabled. 7,831 unique peptide sequences were identified at an FDR of 1%, of which, 7,681 (98%) contained at least one cysteine residue. Peptide intensities from technical replicate analyses were averaged and only peptides with a minimum of 3 valid intensity values (i.e. measured intensity values in three biological replicate experiments) in the hydroxylamine treated set were taken forward for further analysis (4,877 free cysteine containing peptides from 2430 protein groups). Missing values were imputed for control purifications (minus hydroxylamine set) using Perseus so that statistical analysis of the data could be performed. This was necessary because often palmitoylated peptides were robustly identified in the plus hydroxylamine set but were below the limit of detection in the minus hydroxylamine set resulting in missing intensity values. 1,098 palmitoylation sites were significantly enriched (t-testing with a Permutation based FDR of 0.05, S0 = 1) in the plus hydroxylamine versus control sets (Fig. 2a). An additional magnitude of enrichment filter (average ratio plus/minus hydroxylamine >3) was applied to the data and redundant site quantifications were removed leaving a final set of 906 palmitoylation sites mapping to 641 protein groups (Tables 1 and S1). The average fold enrichment in this final set was 33.9 with a median fold enrichment of 10.2. As palmitoylation sites identified using this workflow are inferred by their relative enrichment in hydroxylamine treated versus control samples (same as protein level ABE) and not directly identified, sites chosen for functional experiments should be validated using orthogonal methods.

**Table 1:** Overview and annotation of the brain palmitoyl-proteome

Annotation/statistical enrichment	Significance*	# Sites	# Proteins
Brain palmitoyl-proteome		906	641
Known palmitoylation sites		102 (11.3%)	63 (9.8%)
Previous evidence of palmitoylation	8.80E-34	565 (62.4%)	362 (56.5%)
Contain transmembrane domains	1.06E-88	475 (52.4%)	304 (47.4%)

### Properties of a site-specific brain palmitoyl-proteome

We began by testing whether this final set of statistically enriched palmitoylation sites had features that were different from unmodified cysteine sites (non-enriched cysteine-containing peptides). We annotated the entire dataset for evidence of palmitoylation (UniProt and published proteomics datasets) and membrane association (containing transmembrane helices) and performed Fisher’s exact testing (benjamini Hochberg FDR 0.02) in Perseus. Statistically enriched palmitoylation sites (hydroxylamine Vs control) were significantly more likely to be from proteins with previous evidence of palmitoylation (8.8E-34) and to contain transmembrane domains (1.06E-88) than the background of non-enriched sites (Table 1). 62.3% (565/906 sites) of enriched palmitoylation sites map to proteins with previous evidence of palmitoylation (previous protein level proteomic studies as well as from the literature). These sites map to 377 known palmitoylated proteins representing 58.8% of proteins for which we could confidently identify palmitoylation sites. 52.4% (475/906) of palmitoylation sites are from proteins that contain transmembrane domains (transmembrane domain prediction and UniProt annotation) representing 47.4% (304/641) of proteins with palmitoylation sites. Of the set of 906 palmitoylation sites that we identified using ssABE, 11% (102 palmitoylation sites on 63 proteins) have previously been demonstrated to be palmitoylated at these sites (Tables 1 and S1 (Dataset 1)).

Functional annotation of 641 palmitoylated proteins using a Fisher exact test (Perseus) revealed highly significant enrichment (Benjamini–Hochberg corrected p values) of UniProt keywords such as “Palmitate” (p value 9.46E-41), “Membrane” (p value 5.47E-46) compared to the non-enriched set of proteins (1,789 proteins). Analysis of GO term enrichment in the same set of 641 palmitoylated proteins compared to the mouse brain proteome^24^ using PANTHER reveal enrichment of terms such as “G-protein coupled receptor signaling pathway”, “Ion Transport, “Synaptic transmission” and “Glutamate receptor signaling pathway” compared to the mouse brain proteome (Table 2). The most enriched KEGG pathways included Neuroactive ligand-receptor interaction, Calcium signaling pathway, Long-term depression, and Long-term potentiation. Comparison of the brain palmitoyl-proteome dataset with previous synaptic proteome datasets^25, 26^ reveals that 490 palmitoylation sites were identified on 342 synaptic proteins, 44% of which are integral membrane proteins. This high representation of synaptic proteins in the palmitoyl-proteome is consistent with the important regulatory role of palmitoylation for synaptic ion channels, receptors and PDZ domain containing proteins^27^.

**Table 2:** Functional annotation of the brain palmitoyl-proteome.

Functional annotation enrichment	Significance
**UniProt keywords**	
Membrane	5.74E-46
Palmitate	9.46E-41
**Gene Ontology**	
G-protein coupled receptor signaling pathway	7.07E-29
Ion transport	6.81E-27
Synaptic transmission	4.32E-14
Glutamate receptor signaling pathway	1.65E-05
**KEGG pathways**	
Neuroactive ligand-receptor interaction	3.55E-13
Calcium signaling pathway	2.80E-12
Long-term depression	4.35E-06
Long-term potentiation	8.30E-06

Enrichment of UniProt keywords, Gene Ontology terms and Kegg pathways in the brain palmitoyl-proteome was performed. Significance of enrichment was calculated by Fisher’s exact testing (benjamini Hochberg FDR 0.02).

We annotated all putative palmitoylation sites with data from UniProt to indicate which cysteine residues are known or predicted to form disulfide bonds as well as those involved in other types of thioester bonds. 6.5% of our final set of palmitoylation sites are located on cysteine residues known to or are predicted to form disulfide bonds with a further 2% forming non-lipid thioester bonds (Fig. 2b). Palmitoylation and disulfide bonds are not mutually exclusive with respect to individual sites and there are known examples of cysteine residues that can be palmitoylated or form disulfide bonds^28^. In most cases, ssABE readily differentiated between palmitoylation and disulfide bonds by the quantitative enrichment of peptides in hydroxylamine treated versus control experiments. An example of this is illustrated in Fig. 2c. PLP1 (Myelin proteolipid protein) has six known sites of palmitoylation and four cysteine residues that form two disulfide bonds in the extracellular domain between the third and fourth transmembrane domain. All six palmitoylation sites were identified as free cysteines upon hydroxylamine treatment and peptides containing three disulfide bond forming cysteine residues were identified as carbamidomethylated by ssABE. All peptides containing palmitoylation sites were quantitatively and statistically enriched (permutation based FDR 0.05) in hydroxylamine treated versus control experiments whilst peptides with disulfide bond forming cysteine residues were not enriched. (Fig. 2d). Complete palmitoylation site coverage was also obtained for a t-SNARE involved in the regulation of neurotransmitter release, SNAP25 (Fig. 2e). This protein contains up to four sites of palmitoylation and peptides bearing one, two, three and four palmitoylated residues were identified. The ability to detect all of these forms of palmitoylated SNAP25 is particularly significant because differential palmitoylation of these sites has been shown to control intracellular patterning of SNAP25 between the plasma membrane and the endosomal system^29^.

### Membrane topology of palmitoylation sites

**Figure 3:**
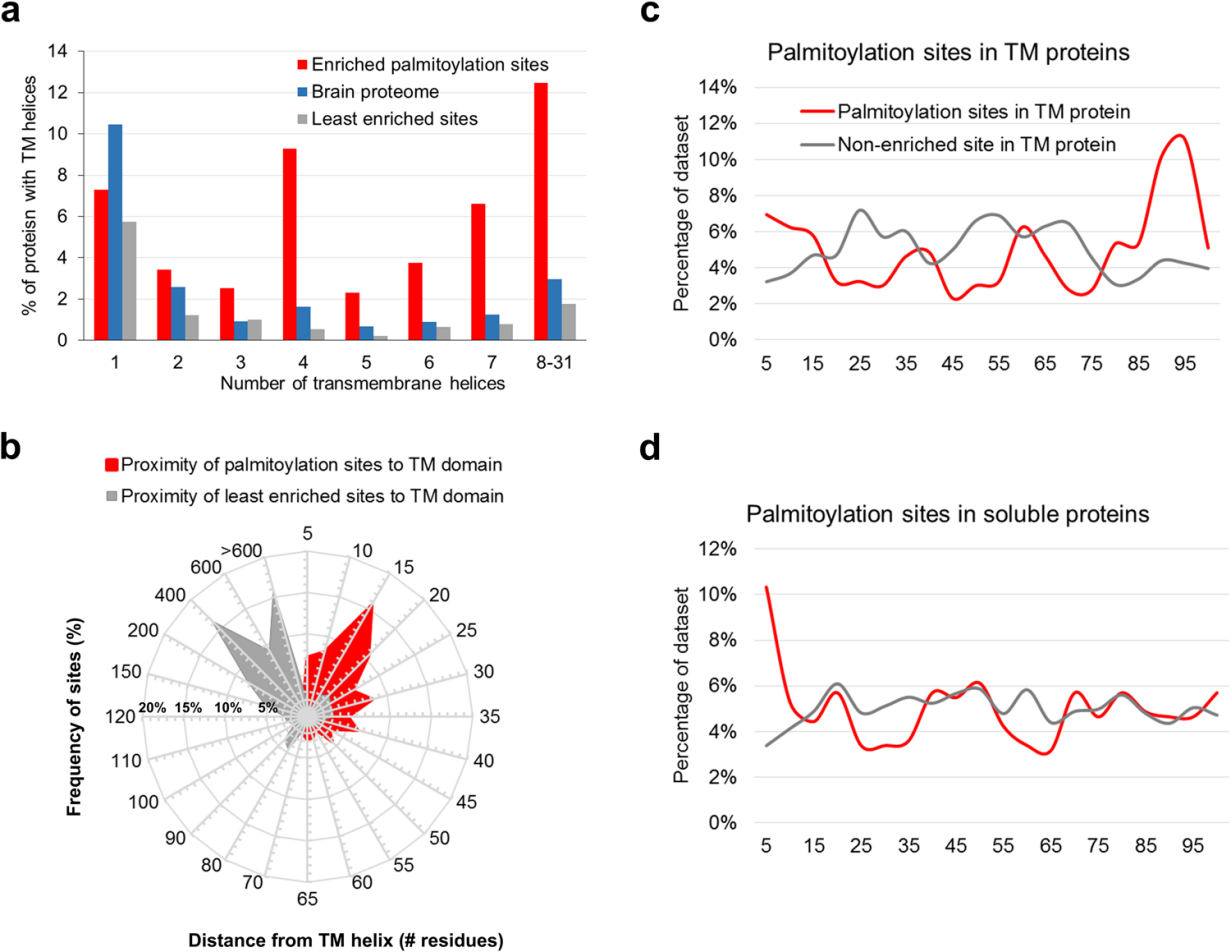
Occurrence and topology of palmitoylation sites in transmembrane proteins. (**a**) The frequency of transmembrane helices in proteins for which we identified palmitoylation sites was compared to that occurring in the set of “least enriched” sites identified by the ssABE method. In general, palmitoylation sites occurred more frequently in multi-membrane spanning proteins with striking enrichment for proteins with 4, 7 and more than 8 transmembrane helices. (**b**) The proximity of palmitoylation sites to transmembrane helices was compared to an equivalent number of the “least enriched” sites identified by the ssABE method. Two distinct distributions are observed with palmitoylation sites most frequently located approximately 15 residues from a transmembrane helix and the “least enriched” sites being most frequent over 400 residues from a transmembrane helix. The position of palmitoylation sites and the set of “least enriched” sites identified by ssABE relative to protein length was compared for transmembrane proteins in (**c**) and soluble proteins in (**d**). Relative positions are expressed as a percentage of the protein length in residues. Palmitoylation sites in transmembrane proteins are most frequently located in the first 10% of proteins (N-terminus) or in the last 20% (C-terminus). Palmitoylated soluble proteins exhibit a different profile with the most frequent position of palmitoylation sites located in the first 10% of the proteins (extreme N-terminus) but without the C-terminal distribution spike observed for palmitoylated transmembrane proteins.

There was a clear enrichment for palmitoylation sites on membrane proteins; 52.4% (475/906 sites) of the final ssABE enriched set (by average +/− hydroxylamine ratio) of palmitoylation sites were in proteins with transmembrane helices. Of the total set of 641 palmitoylated proteins, 47.4% (304) contained at least one transmembrane helix compared to a frequency of 26% predicted for the entire human proteome^30^. We investigated the distribution of sites in proteins with increasing numbers of transmembrane helices (Fig. 3a). We observed that the enriched set of palmitoylation sites were more likely to be present on multi-membrane spanning proteins than the least enriched (+/− hydroxylamine treatment) set of sites. We have repeated this analyses using a recent brain proteome dataset of approx. 10,000 proteins detected in mouse brain tissue using LC-MS/MS^24^ and observed a 4.64-fold enrichment of proteins with four or more transmembrane helices in the palmitoyl- proteome set compared to the mouse brain proteome. Striking enrichment is particularly evident for proteins with 4, 7 and more than 8 transmembrane helices. Analysis of Gene Ontology terms associated with the set of palmitoylated proteins with 8 or more transmembrane domains revealed a strong enrichment for proteins involved in “Transmembrane Transport” (adjP = 1.44e-35, hypergeometric test with BH adjustment), particularly those involved in sodium ion transport (Sodium channels), neurotransmitter transport (e.g. glutamate, GABA, dopamine) as well as various amino acid and small molecular transporters. Another over-represented set of proteins contain 7 transmembrane helices corresponding to palmitoylation of a large number of GPCRs; the biological process “G-protein coupled receptor activity” was significantly enriched in this set (adjP = 3.46e-26, hypergeometric test with Benj. Hoch. adjustment). In fact, 63 palmitoylation sites were identified on 42 GPCRs (as defined by the presence of the interpro domain “GPCR” characteristic of this class of proteins), 36 of which belong to the rhodopsin-like GPCR domain family. This domain was highly enriched in the set of 906 palmitoylation sites compared to non-enriched (+/− hydroxylamine) sites (Benj. Hoch. FDR 3.3665E-29). 48% (20/42) of these GPCRs have previously been shown to be palmitoylated and 19 known palmitoylation sites were identified. Next, we looked to see if any patterns were evident in terms of the topology of palmitoylation of GPCRs and found that the majority (88%) GPCRs had at least one palmitoylation site N-terminal to their last transmembrane domain, confirming the trend observed form numerous studies of individual GPCR palmitoylation (Figure S1). Some GPCR’s have additional palmitoylation sites but they all occur after the third transmembrane domain. Palmitoylated proteins with four transmembrane helices were enriched for “potassium ion transmembrane transport” e.g. voltage gated potassium channels (Benj. Hoch. FDR 8.45e-06) and ion gated channel activity (Benj. Hoch. FDR 1.52e-14), examples of which include GABA and Glutamate receptors. Finally, the enrichment of proteins with four transmembrane helices can also be accounted for by the presence of several ZDHHC PATs which are themselves palmitoylated.

We next investigated the proximity of palmitoylation sites to transmembrane helices in proteins. We measured the distance between palmitoylation sites and the nearest transmembrane helix (first or last residue of the membrane spanning domain) and plotted the frequency of palmitoylation sites at increasing intervals from transmembrane domains (Fig. 3b). The proximity between sites and transmembrane domains clearly segregates those sites that are identified as palmitoylated and those that are not by ssABE. The most frequent position of palmitoylation sites is 15 residues from a transmembrane domain interface and 74% of all palmitoylation sites identified on transmembrane proteins were within 50 residues of a transmembrane domain. In order to investigate whether palmitoylation of integral membrane proteins and soluble proteins differ in terms of their topological location we plotted the relative position of palmitoylation sites and the least enriched sites (by ssABE) in terms of their relative position expressed as a percentage of protein length (Fig. 3c,d). Palmitoylation sites in membrane proteins are most frequent at their extreme N-terminus and also their C-terminus, a characteristic feature of GPCRs. Soluble proteins are most frequently palmitoylated at their N-terminus consistent with that observed for well-known soluble proteins such as PSD-95 and Grip1.

### Concurrent identification of palmitoylation and phosphorylation sites

The relationship between different post-translational modifications on the same protein is often poorly understood but recent examples of regulatory crosstalk between palmitoylation and phosphorylation^31–33^ prompted analysis of our brain palmitoyl-proteome data with the aim of identifying peptides that were co-modified. Raw data was analyzed using MaxQuant with ser/thr/tyr phosphorylation set as a variable modification. 156 PSMs were identified that contained both potential palmitoylation and phosphorylation sites in the same spectrum with a posterior error probability cut off of 0.01 (Table S2 (Dataset 2)). Of these, 46 identified palmitoylation sites (Table S2 and Figure S2) mapped to proteins that we had already identified in the brain palmitoyl-proteome and 35 identified palmitoylation sites that we had already identified in the brain palmitoyl proteome, i.e. independently identified as significantly enriched palmitoylation sites from the same raw data files. An additional 4 sites that were identified as potential palmitoylation sites but were not statistically enriched in +/− hydroxylamine treatment were also identified on phosphopeptides in this re-analysis of the data. Of the 46 phosphorylation sites on peptides co-modified with palmitoylation, 23 have been previously reported (PhosphoSitePlus).

**Figure 4:**
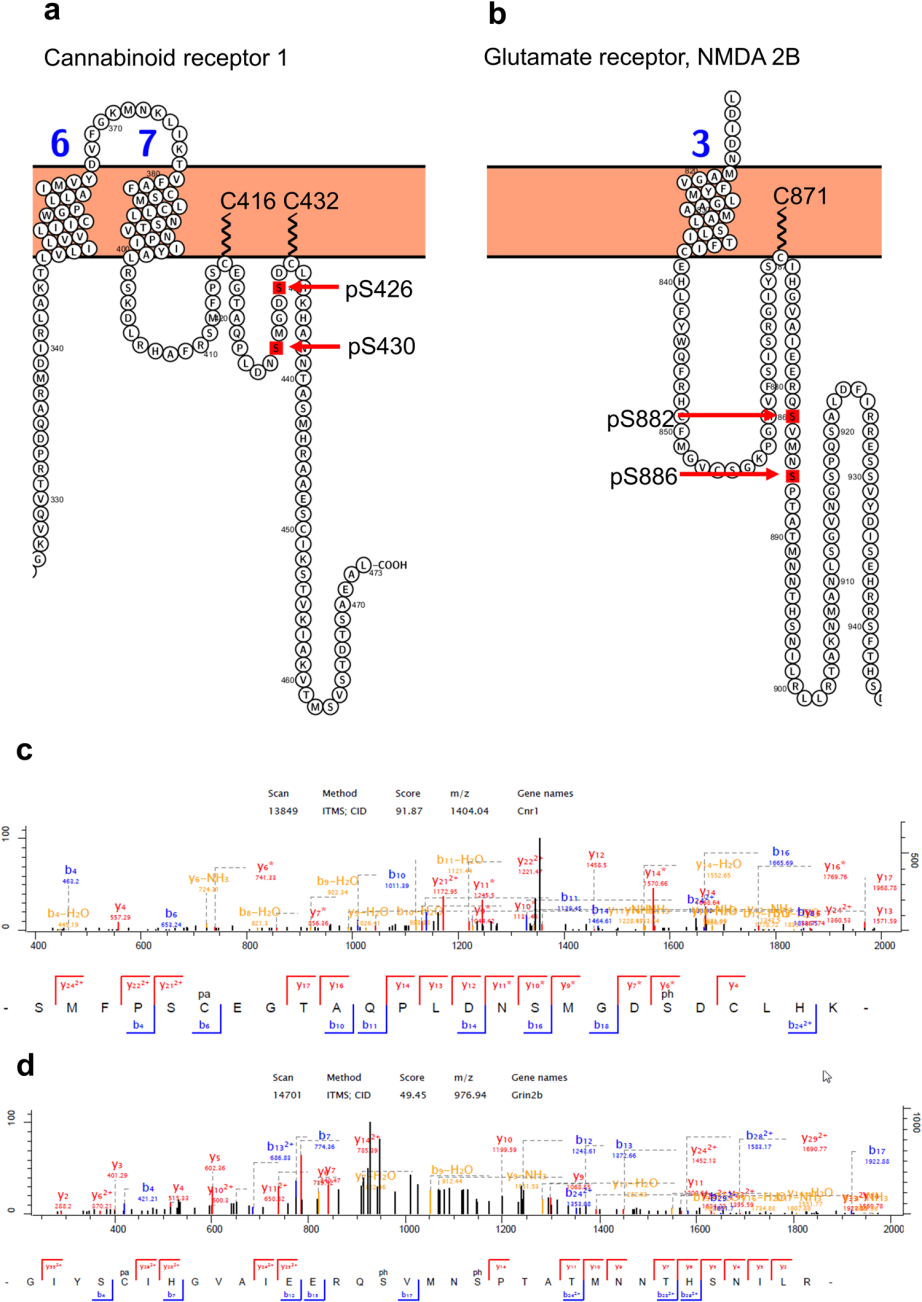
Concurrent identification of palmitoylation and phosphorylation sites. Examples of potential interplay between palmitoylation and phosphorylation are shown in (**a**) Cannabinoid receptor 1, (**b**), Glutamate receptor, NMDA 2B **(c)** associated fragmentation spectra shown in (**d**,**e**), respectively. 46 phosphorylated (with 23 known sites of phosphorylation) and palmitoylated peptides (co-modified) were identified and these sites of palmitoylation were also independently identified in the brain palmitoyl proteome.

Examples of two of these cases are illustrated in Fig. 4. We identified a peptide mapping to the c-terminal cytoplasmic domain of the Cannabinoid receptor 1 that identified Cys416 as palmitoylated (Fig. 4a). Mutation of this site functionally impairs membrane targeting and signaling of the receptor by reducing recruitment at both plasma membrane and lipid rafts and decoupling the receptor from G-proteins^34^. This same peptide also identified two phosphorylation sites (Ser426 and Ser430) which when phosphorylated by a G-protein-coupled receptor kinase (GRK) results in receptor desensitization and regulates an acute response to, tolerance to, and dependence on cannabinoids^35^. An additional novel palmitoylation site was identified at Cys432; palmitoylation at both sites could form a double loop topology in the cytoplasmic domain which could create a protein interaction site or could alter the conformation of the protein to regulate phosphorylation at Ser426 and S430. The interplay between phosphorylation and palmitoylation at these sites is not fully understood but given their individual functions and proximity, it is possible that they interact to form a regulatory module to control receptor activity. We identified a similar arrangement of palmitoylation and phosphorylation sites for the Glutamate receptor, NMDA2B subunit (Fig. 4b). These receptors are palmitoylated at two distinct sites^36^ and we have identified a known site in cluster 1 (Cys871) which regulates the phosphorylation of nearby tyrosine phosphorylation sites to enhance stable surface expression of the receptor. We identified two additional known phosphorylation sites (Ser882 and Ser886)^36–39^ on the peptide identifying Cys871 as palmitoylated. The function of these two phosphorylation sites is unknown but proximity to Cys871 may allow a functional interaction between phosphorylation and palmitoylation at these sites.

### Potential palmitoylation motifs

**Figure 5:**
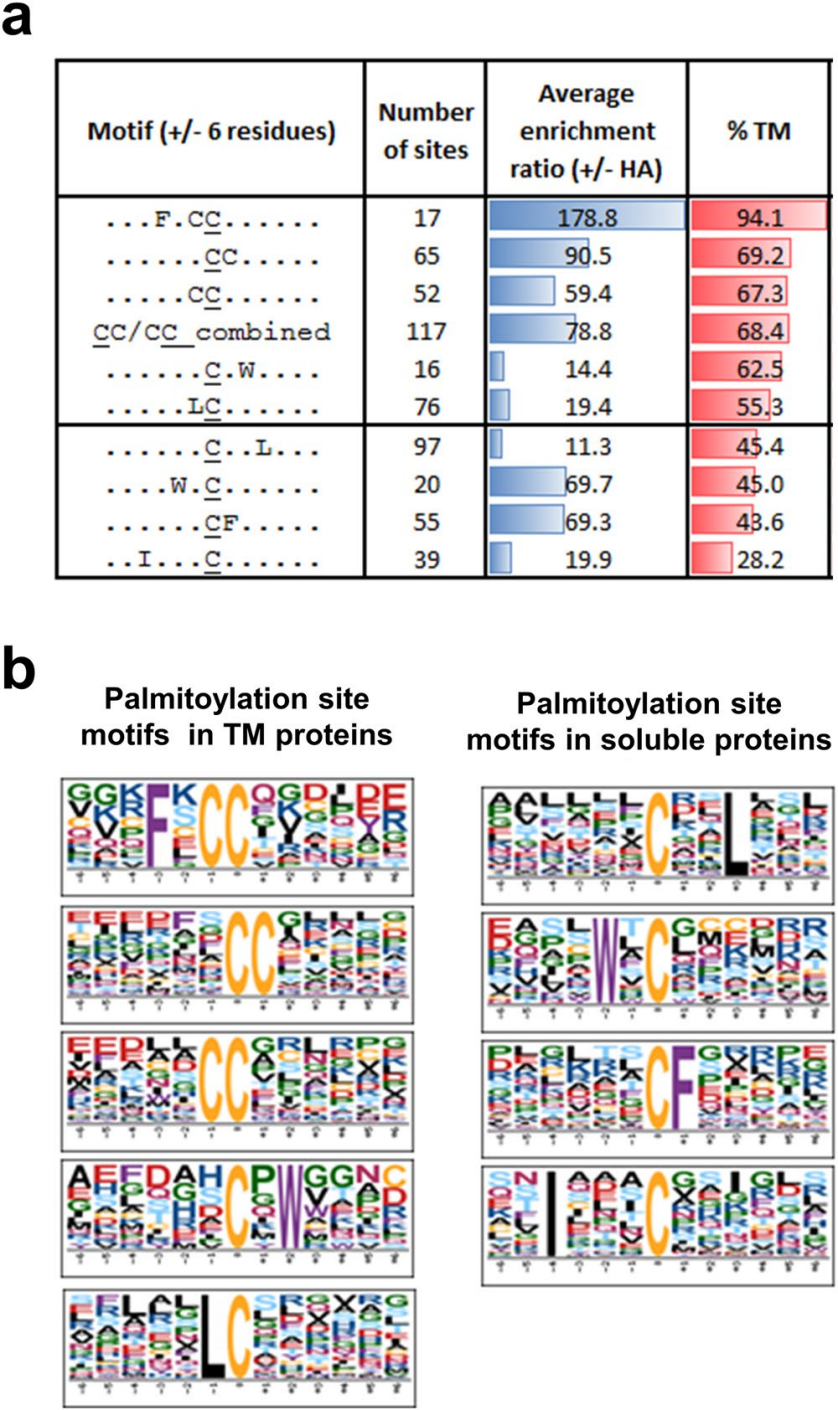
Palmitoylation site motifs distinguish palmitoylation of transmembrane and soluble proteins. (**a**,**b**) Motif-x was used to identify significantly enriched sequence motifs in sequences +/− 6 residues in the set of 906 enriched palmitoylation sites compared to the mouse proteome. Re-analysis of palmitoylation sites from proteins with transmembrane helices and those that do not (soluble proteins) revealed that these two types of palmitoylated proteins have distinct enriched sequence motifs with transmembrane proteins containing mainly di-cysteine containing motifs and soluble proteins containing single cysteine residues and leucine, tryptophan, phenylalanine and isoleucine and specific positions.

A major motivation for developing a method that permits large-scale analysis of palmitoylation sites is the ability to explore sequence features and motifs in the vicinity of this modification and to improve prediction of palmitoylation sites. Motif-x was used to identify overrepresented motifs in sequences surrounding palmitoylation sites (+/− 6 residues) compared to the mouse proteome. Although this approach would be akin to taking a collection of phosphorylation sites and looking for motifs without any knowledge of kinase substrate specificity, we hypothesized that the most prominent features that distinguish sites modified by groups of PATs might be evident. At a significance cut-off of 0.001, 9 distinct motifs were evident in the set of 906 palmitoylation sites (Fig. 5); dicysteine motifs and single cysteine motifs with hydrophobic residues (Leucine, Isoleucine, Tryptophan and Phenylalanine) at specific positions relative to palmitoylation sites. Annotation of these different motifs with the presence or absence of transmembrane helices in proteins associated with them revealed that some motifs (FxCC) were almost exclusively in transmembrane proteins whilst others (IxxxC) were predominantly in soluble proteins. Repetition of this analysis on sites within transmembrane proteins and soluble proteins separately, revealed that the original 9 motifs segregated into two groups on the basis of the occurrence of transmembrane domains in associated proteins. Palmitoylation sites on transmembrane proteins were enriched for di-cysteine motifs with CC* and C*C (*denotes palmitoylation site) accounting for 25% of all sites in this category and the motif FxCC having the highest enrichment in ssABE experiments, highest occurrence of transmembrane domains and the highest content of previously known palmitoylated proteins (Fig. 5). Soluble proteins only contained motifs containing single cysteines and hydrophobic amino acids within 4 residues and the most common motifs being CxxL and CF.

### Palmitoylation of palmitoyl acyltransferases

**Figure 6:**
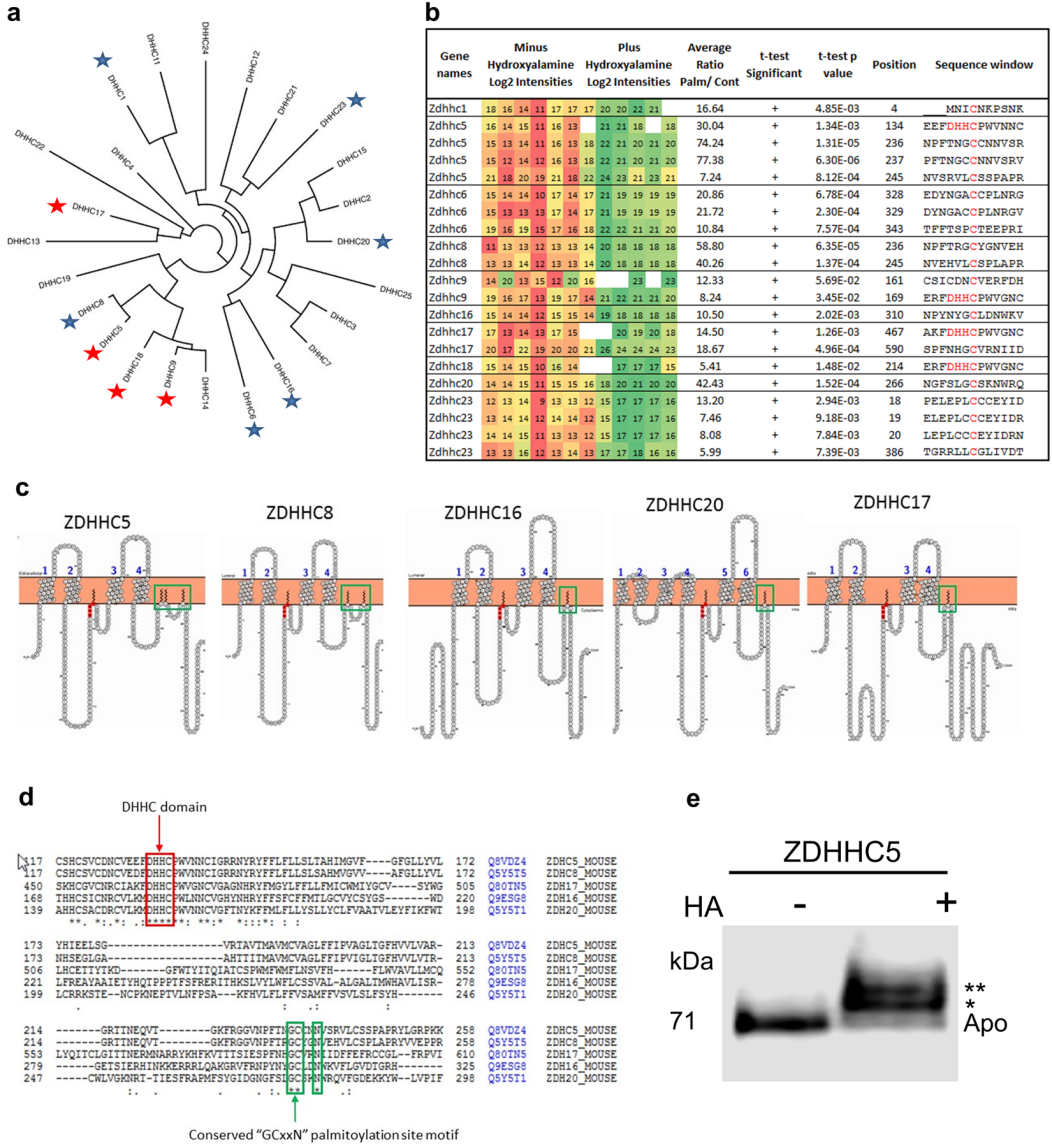
Palmitoylation of protein acyltransferases. (**a**) 21 palmitoylation sites were identified on 10 (starred) out of 23 known DHHC-domain containing protein acyltransferases. (**b**) Relative intensities of peptides identifying these modifications are shown in +/− hydroxylamine treated sets. All detected potential palmitoylation sites for PATs were significantly enriched over control purifications in ssABE experiments. (**c**,**d**) Palmitoylated active site intermediates were detected for 4 PATs (starred red in (**a**)) and additional non-active site palmitoylation was found for 9 PATs, a subset of which was in a conserved “GC*xxN” motif in 5 PATs (**c**). (**e**) PEG switch assay of endogenous mouse brain DHHC5. Band shifts corresponding to the addition of 5 kDa PEG groups to sites of palmitoylation sensitive to hydroxylamine HA) treatment allow the detection of at least two palmitoylated forms of DHHC5 thus confirming that it is also palmitoylated at sites other than the active site cysteine residue. An uncropped image of this PEG switch blot is presented in Figure S3.

We identified 21 sites of palmitoylation on 10 DHHC domain-containing palmitoyl-acyltransferases (Fig. 6) representing 43% of all known palmitoyl-acyltransferases^1^ and likely the majority of those expressed in the brain. We identified the S-palmitoyl cysteine intermediate that occurs in the active site cysteine residue in the “DHHC*” domain for 4 PATs (ZDHHC5, 9, 17 &18). Whilst, active site palmitoylation has been directly detected in experiments targeted to individual PATs^40^, our approach facilitates unbiased site-specific detection of active site palmitoylation of PATs by mass spectrometry. PAT active sites are rapidly autopalmitoylated and then either the palmitoyl group is transferred to substrate proteins or it is hydrolyzed to release free fatty acid^41^. In either case, active site palmitoylation is continuously turned over but the enzyme exists in a permanently palmitoylated state in the absence of substrates^41^. ZDHHC5 regulates AMPA receptor trafficking by palmitoylation of the glutamate receptor interacting protein, GRIP1^42^, membrane association of BK channel STREX variant^43^, is regulated by induction of neuronal differentiation^44^, interacts with PDZ domain containing scaffolding proteins such as PSD-95 and plays a role in learning and memory^45^. ZDHHC17 (Hip14) was first shown to palmitoylate huntingtin protein and regulate its trafficking a process that is affected by polyglutamine tract expansion in Huntington’s disease^46^. Since then it has been shown to regulate SNAP25/23^47^ and STREX^43^ membrane association and its absence in knockout mice causes alterations in synaptic plasticity and impaired hippocampal memory formation^48^. ZDHHC9 palmitoylates Ras proteins^49^ and STREX^43^ and the least well characterized is ZDHHC18 which is capable of palmitoylating H-Ras and Lck^1^. These four PATs for which we have detected active site palmitoylation are likely to represent the most abundant or active members of this enzyme family in the brain and include two which have been shown to be very important for neuronal specific processes.

In addition, we have also identified 17 sites of palmitoylation located C-terminal to the active site in several PATs with a site in 5 enzymes (ZDHHC5, 8, 16, 17 & 20) clustered to a single conserved cysteine in a “GCxxN” motif (Fig. 6c). A recent study identified a high confidence palmitoylation site in this region of ZDHHC5^19^ and we have now extended and experimentally confirmed that this conserved site is palmitoylated in four other family members. In ZDHHC5 and ZDHHC8, we detect additional sites C-terminal to this, with 8 mapping to the previously described CCX_7–13_C*(S/T) motif, where C* is palmitoylated^19^. In order to confirm that PATs are indeed multiply palmitoylated *in vivo*, we performed a PEG-switch assay^50^ using a mouse brain extract and found that DHHC5 protein exists in a predominantly palmitoylated form with evidence for multiply palmitoylated states (Fig. 6e) as evidenced by multiple hydroxylamine dependent band shifts. The functional consequences of these additional palmitoylation sites are unknown but they may modulate the conformation of the cytoplasmic tail of these PATs to create binding sites for substrates or indeed regulatory proteins.

## Conclusions

We have developed a workflow that builds upon standard acyl-biotin exchange assays to permit large scale analysis of palmitoylation sites. This has enabled the first global analysis of the brain palmitoyl-proteome with palmitoylation site resolution and has allowed us to investigate sequence features surrounding palmitoylation sites. We find that integral membrane proteins and soluble proteins are palmitoylated but that integral membrane proteins are disproportionately enriched given that they contribute over half of the dataset but represent just over a quarter of the proteome. Differences in the topology and potential palmitoylation motifs suggest that different PATs may modify different sets of proteins and that palmitoylation may have diverse functional consequences. Clearly, palmitoylation of soluble proteins aids membrane tethering whilst palmitoylation of integral membrane proteins may have a more subtle effect. Perhaps, tethering of intracellular loops can regulate integral membrane function by changing accessibility of protein interactors or enzymes mediating other PTMs. Palmitoylation near transmembrane helices alters protein structure by causing tilting of membrane spanning domains and to regulate protein sorting to lipid rafts. As well as allowing questions to be asked about the exact positioning of palmitoylation sites, ssABE permitted the identification of peptides co-modified by both palmitoylation and phosphorylation. Given the interplay of these two modifications that has been established for a few examples, the identification of co-modified peptides using ssABE should allow this phenomenon to be investigated in more detail. The ability to identify and quantify active site palmitoylation of PATs using ssABE was unexpected but adds yet another dimension to the approach. This permits the most active/abundant PATs to be identified, giving a first step toward narrowing down the list of PATs that can modify substrates in that sample. Finally, given that ssABE is quantitative, it could be used in perturbation experiments to quantify differential palmitoylation with site-specific resolution. The combination of ssABE with perturbations or stimulation paradigms offers the potential to dissect the role of palmitoylation associated with specific biological questions and the opportunity to expand our understanding of this class of post-translational modification.

## Experimental procedures

### Protein extraction and reduction and alkylation

Mouse forebrain tissue was solubilized in the following extraction buffer (4% SDS, 0.1 M Tris pH 8.5, 2 ug/μl aprotinin/leupeptin, 0.5 mM PMSF, 20 μM ZnCl, 5 mM EDTA, 50 mM TCEP, 25 mM iodoacetamide). 8.5 ml of extraction buffer was used per forebrain. Samples were homogenized 25 times dounce homogenizer and heated at 70 °C for 20 min and DNA was sheared by passing the extract through fine gauge needle 10 times. Insoluble material was removed by centrifugation and the supernatant was retained. Additional iodoacetamide was added to bring the concentration to 50 mM and urea was added to a final concentration of 8 M and was incubated in the dark for 3 hours at room temperature to allow alkylation to proceed to completion.

### Sample clean-up

Two aliquots (2 mg each) were transferred to two prewashed (8 M Urea/100 mM Tris pH 8) amicon ultra 30 kD MWCO spin columns (Millipore) and were centrifuged to begin buffer exchange. The samples were buffer exchanged with the addition of 2 mL 8 M Urea/100 mM Tris pH 7.4 and centrifugation, five times.

### Acyl-biotin exchange and sample clean-up

HPDP-biotin solubilized in 20 μl N,N-dimethylforamide was added to the sample in the upper chamber of the amicon ultra unit to achieve a final concentration of 1 mM biotin. Hydroxylamine pH 7.4 was added to the +HA sample to a final concentration of 0.7 M and 100 mM Tris pH 7.4 was added to the control (−HA) sample. Hydroxylamine treatment and biotin labelling of newly released free cysteine residues was allowed to proceed for 1 hr at room temperature and then samples were buffer exchanged with the addition of 2 mL 8 M Urea/100 mM Tris pH7.4 and centrifugation, four times and a final wash with 2 mL 8 M Urea/100 mM ammonium bicarbonate.

### Tryptic digestion and peptide collection

The samples were diluted to a final concentration of 1 M Urea and were digested with Trypsin Gold (Promega) at an enzyme substrate ratio of 1:50 for 3 hours at 37 °C. Peptides were collected from the upper chamber and the chamber membrane was washed with 2 ml 2× LB buffer (100 mM Tris, 300 mM NaCl, 10 mM EDTA, pH 7.4). After centrifugation the collected peptides were pooled and stored overnight at −20 °C.

### Purification of biotinylated peptides

100 μl bed volume of streptavidin-agarose resin was washed with LB buffer (3 × 1 ml) and was added to the peptide samples and incubated for 1 hr at room temp. The supernatant was washed by gentle centrifugation and the resin was washed with 10 ml of wash buffer (0.1% SDS, 0.2% Tx-100, 50 mM Tris pH 7.4, 150 mM NaCl), incubated for 10 min and repeated. The resin was transferred to a mini-column and washed 2 × 10 ml wash buffer (2 M Urea/100 mM Tris pH 7.4) using a syringe, then with 2 ml H_2_O. Peptides were eluted by the addition of 50 μl 10 mM TCEP and incubation for 10 min at 37 °C, this was repeated and eluates were combined and acidified by the addition of formic acid.

### LC-MS/MS analysis

Peptides from purifications were split and each analyzed by 2 replicate LC-MS/MS analyses. Samples were analyzed using an Ultimate 3000 RSLC Nano LC System (Dionex) coupled to an LTQ Orbitrap Velos hybrid mass spectrometer (Thermo Scientific) equipped with an Easy-Spray (Thermo Scientific) ion source. Peptides were desalted on-line using a capillary trap column (Acclaim Pepmap100, 100 μm, 75 μm × 2 cm, C18, 5 μm (Thermo Scientific)) and then separated using 120/180 min RP gradient (4–30% acetonitrile/0.1% formic acid) on an Acclaim PepMap100 RSLC C18 analytical column (2 μm, 75 μm id x 50 cm, (Thermo Scientific)) with a flow rate of 0.3 μl/min. The mass spectrometer was operated in standard data dependent acquisition mode controlled by Xcalibur 2.2. The instrument was operated with a cycle of one MS (in the Orbitrap) acquired at a resolution of 60,000 at m/z 400, with the top 15 most abundant multiply-charged (2 + and higher) ions in a given chromatographic window were subjected to CID fragmentation in the linear ion trap. An FTMS target value of 1e6 and an ion trap MSn target value of 10000 were used. Dynamic exclusion was enabled with a repeat duration of 30 s with an exclusion list of 500 and exclusion duration of 60 s. Lock mass of 401.922 was enabled for all experiments.

### Mass Spectrometry data analysis

Data was analyzed using MaxQuant version 1.4.1.2^23^. MaxQuant processed data was searched against a UniProt (downloaded October 2013) mouse sequence database (52654 sequences) using the following search parameters: trypsin with a maximum of 2 missed cleavages, 7 ppm for MS mass tolerance, 0.5 Da for MS/MS mass tolerance, with acetyl (Protein N-term) and oxidation (M) as variable modifications and carbamidomethyl (C) as a fixed modification. Palmitoylation sites were reported and localized in peptide sequences by the use of a loss of 57.02146 (i.e. unmodified cysteine) as only those cysteines targeted by acyl-biotin exchange should be unmodified, all other cysteines should be alkylated. A protein FDR of 0.01 and a peptide FDR of 0.01 were used for identification level cut offs. Class I palmitoylation sites were defined with a localization probability of >0.75 and a score difference of >5. Match between runs with a 2 minute retention time window was enabled and peptide intensity values calculated by MaxQuant were used to quantify enrichment of peptides in +/− hydroxylamine treated samples (6 biological replicates and 2 technical replicates, each). Data was filtered so that at least 3 valid intensity values were present (3/6 biological replicates) in hydroxylamine treated samples and log2 transformed. Missing values in control experiments were imputed using Perseus (1.4.1.3) and two sample t-testing was performed with a permutation based FDR calculation in Perseus. Peptides enriched within an FDR of 0.05 with at least a 3-fold change were defined as being enriched hydroxylamine treated samples and therefore identifying peptides with putative sites of palmitoylation. Analysis of enrichment of UniProt keywords in the set of palmitoylated proteins compared to the non-enriched set was performed using Perseus with a Fisher exact test and a Benjamini-Hochberg FDR of 0.02. Analysis of GO term enrichment in the set of palmitoylated proteins compared to the mouse brain proteome^24^ was performed using a PANTHER^51^ overrepresentation test with Bonferroni correction for multiple testing. Enrichment of KEGG pathways in the same set of palmitoylated proteins compared to the mouse brain proteome was performed using WebGestalt^52^ with Benjamini-Hochberg correction for multiple hypothesis testing.

### PEG-switch assays

PEG switch assays were performed as described^50^ with some minor modifications. Whole mouse forebrain was homogenized in 4% SDS, 50 mM Tris pH7.2 and 1 mM EDTA. Lysates were heated at 70 °C for 20 minutes and DNA was sheared by passing the lysates through a fine gauge needle. After cooling, HEPES buffer and maleimide were both added to 100 mM final concentration and samples were incubated at 40 °C for 3 hours with shaking to block all free cysteines. Protein was precipitated with 4× volume of ice-cold acetone, and the pellet was washed 5 times with 80% acetone to remove any excess maleimide. Pellets were re-suspended in 1% SDS, 1 mM EDTA and 50 mM Tris pH 7.2 and split into +/− hydroxylamine samples. To the +HA sample 2 M HA pH 7.2/50 mM Tris pH 7.2 was added to a final concentration of 1 M HA, to the –HA sample, the same volume of 50 mM Tris pH 7.2 was added. Samples were left at room temperature for 1 hour with end over end rotation. Acetone precipitation of protein was performed as described above. Pellets were re-suspended in 1% SDS/50 mM Tris pH 7.2, and mPEG 5 kDa was added to 5 mM final concentration. Samples were incubated at 25 °C for 1 hour with shaking. Protein was acetone precipitated as described above. Pellets were re-suspended in 2.5% SDS/50 mM Tris, before western blotting using an anti-ZDHHC5 Antibody (Atlas Antibodies (HPA014670)).

## Electronic supplementary material

Supplementary Figures

Dataset 1

Dataset 2

## References

[1] Fukata M, Fukata Y, Adesnik H, Nicoll RA, Bredt DS (2004). Identification of PSD-95 palmitoylating enzymes. Neuron.

[2] Duncan JA, Gilman AG (1998). A cytoplasmic acyl-protein thioesterase that removes palmitate from G protein alpha subunits and p21(RAS). J Biol Chem.

[3] Dekker FJ (2010). Small-molecule inhibition of APT1 affects Ras localization and signaling. Nat Chem Biol.

[4] Camp LA, Hofmann SL (1993). Purification and properties of a palmitoyl-protein thioesterase that cleaves palmitate from H-Ras. J Biol Chem.

[5] Lin DT, Conibear E (2015). ABHD17 proteins are novel protein depalmitoylases that regulate N-Ras palmitate turnover and subcellular localization. eLife.

[6] Magee AI, Gutierrez L, McKay IA, Marshall CJ, Hall A (1987). Dynamic fatty acylation of p21N-ras. The EMBO journal.

[7] Topinka JR, Bredt DS (1998). N-terminal palmitoylation of PSD-95 regulates association with cell membranes and interaction with K+ channel Kv1.4. Neuron.

[8] Jeffries O, Tian L, McClafferty H, Shipston MJ (2012). An electrostatic switch controls palmitoylation of the large conductance voltage- and calcium-activated potassium (BK) channel. J Biol Chem.

[9] Roth AF (2006). Global analysis of protein palmitoylation in yeast. Cell.

[10] Martin BR, Cravatt BF (2009). Large-scale profiling of protein palmitoylation in mammalian cells. Nat Methods.

[11] Drisdel RC, Green WN (2004). Labeling and quantifying sites of protein palmitoylation. Biotechniques.

[12] Greaves J, Chamberlain LH (2011). DHHC palmitoyl transferases: substrate interactions and (patho)physiology. Trends in biochemical sciences.

[13] Hang HC (2007). Chemical probes for the rapid detection of Fatty-acylated proteins in Mammalian cells. J Am Chem Soc.

[14] Jones, M. L., Collins, M. O., Goulding, D., Choudhary, J. S. & Rayner, J. C. Analysis of protein palmitoylation reveals a pervasive role in Plasmodium development and pathogenesis. *Cell Host Microbe* **12**, 246–258, doi:S1931-3128(12)00231-4[pii]10.1016/j.chom.2012.06.005 (2012). 10.1016/j.chom.2012.06.005 PMC3501726 22901544

[15] Kang R (2008). Neural palmitoyl-proteomics reveals dynamic synaptic palmitoylation. Nature.

[16] Wan J (2013). Tracking brain palmitoylation change: predominance of glial change in a mouse model of Huntington’s disease. Chemistry & biology.

[17] Hayashi T, Rumbaugh G, Huganir RL (2005). Differential regulation of AMPA receptor subunit trafficking by palmitoylation of two distinct sites. Neuron.

[18] Zuckerman DM, Hicks SW, Charron G, Hang HC, Machamer CE (2011). Differential regulation of two palmitoylation sites in the cytoplasmic tail of the beta1-adrenergic receptor. J Biol Chem.

[19] Yang W, Di Vizio D, Kirchner M, Steen H, Freeman MR (2010). Proteome scale characterization of human S-acylated proteins in lipid raft-enriched and non-raft membranes. Mol Cell Proteomics.

[20] Forrester MT (2011). Site-specific analysis of protein S-acylation by resin-assisted capture. Journal of lipid research.

[21] Wisniewski, J. R., Zougman, A., Nagaraj, N. & Mann, M. Universal sample preparation method for proteome analysis. *Nat Methods* **6**, 359–362, doi:nmeth.1322 [pii] 10.1038/nmeth.1322 (2009). 10.1038/nmeth.1322 19377485

[22] Zielinska, D. F., Gnad, F., Wisniewski, J. R. & Mann, M. Precision mapping of an *in vivo* N-glycoproteome reveals rigid topological and sequence constraints. *Cell* **141**, 897–907, doi:S0092-8674(10)00386-7[pii]10.1016/j.cell.2010.04.012 (2010). 10.1016/j.cell.2010.04.012 20510933

[23] Cox J, Mann M (2008). MaxQuant enables high peptide identification rates, individualized p.p.b.-range mass accuracies and proteome-wide protein quantification. Nat Biotechnol.

[24] Sharma K (2015). Cell type- and brain region-resolved mouse brain proteome. Nat Neurosci.

[25] Collins, M. O. *et al*. Molecular characterization and comparison of the components and multiprotein complexes in the postsynaptic proteome. *J Neurochem* **97** Suppl 1, 16–23, doi:JNC3507 [pii] 10.1111/j.1471-4159.2005.03507.x (2006). 10.1111/j.1471-4159.2005.03507.x 16635246

[26] Fernandez, E. *et al*. Targeted tandem affinity purification of PSD-95 recovers core postsynaptic complexes and schizophrenia susceptibility proteins. *Mol Syst Biol* **5**, 269, doi:msb200927 [pii]10.1038/msb.2009.27 (2009). 10.1038/msb.2009.27 PMC2694677 19455133

[27] Thomas GM, Hayashi T (2013). Smarter neuronal signaling complexes from existing components: how regulatory modifications were acquired during animal evolution: evolution of palmitoylation-dependent regulation of AMPA-type ionotropic glutamate receptors. BioEssays: news and reviews in molecular, cellular and developmental biology.

[28] Antinone SE (2013). Palmitoylation of superoxide dismutase 1 (SOD1) is increased for familial amyotrophic lateral sclerosis-linked SOD1 mutants. J Biol Chem.

[29] Greaves J, Chamberlain LH (2011). Differential palmitoylation regulates intracellular patterning of SNAP25. J Cell Sci.

[30] Fagerberg L, Jonasson K, von Heijne G, Uhlen M, Berglund L (2010). Prediction of the human membrane proteome. Proteomics.

[31] Tian L (2008). Palmitoylation gates phosphorylation-dependent regulation of BK potassium channels. Proc Natl Acad Sci USA.

[32] Charych EI, Jiang LX, Lo F, Sullivan K, Brandon NJ (2010). Interplay of palmitoylation and phosphorylation in the trafficking and localization of phosphodiesterase 10A: implications for the treatment of schizophrenia. The Journal of neuroscience: the official journal of the Society for Neuroscience.

[33] Gauthier-Kemper A (2014). Interplay between phosphorylation and palmitoylation mediates plasma membrane targeting and sorting of GAP43. Molecular biology of the cell.

[34] Oddi S (2012). Effects of palmitoylation of Cys(415) in helix 8 of the CB(1) cannabinoid receptor on membrane localization and signalling. British journal of pharmacology.

[35] Morgan DJ (2014). Mutation of putative GRK phosphorylation sites in the cannabinoid receptor 1 (CB1R) confers resistance to cannabinoid tolerance and hypersensitivity to cannabinoids in mice. The Journal of neuroscience: the official journal of the Society for Neuroscience.

[36] Hayashi T, Thomas GM, Huganir RL (2009). Dual palmitoylation of NR2 subunits regulates NMDA receptor trafficking. Neuron.

[37] Ghafari M (2012). Mass spectrometrical identification of hippocampal NMDA receptor subunits NR1, NR2A-D and five novel phosphorylation sites on NR2A and NR2B. J Proteome Res.

[38] Trinidad JC (2012). Global identification and characterization of both O-GlcNAcylation and phosphorylation at the murine synapse. Mol Cell Proteomics.

[39] Huttlin EL (2010). A tissue-specific atlas of mouse protein phosphorylation and expression. Cell.

[40] Gottlieb CD, Zhang S, Linder ME (2015). The Cysteine-rich Domain of the DHHC3 Palmitoyltransferase Is Palmitoylated and Contains Tightly Bound Zinc. J Biol Chem.

[41] Mitchell DA, Mitchell G, Ling Y, Budde C, Deschenes RJ (2010). Mutational analysis of Saccharomyces cerevisiae Erf2 reveals a two-step reaction mechanism for protein palmitoylation by DHHC enzymes. J Biol Chem.

[42] Thomas GM, Hayashi T, Chiu SL, Chen CM, Huganir RL (2012). Palmitoylation by DHHC5/8 targets GRIP1 to dendritic endosomes to regulate AMPA-R trafficking. Neuron.

[43] Tian L, McClafferty H, Jeffries O, Shipston MJ (2010). Multiple palmitoyltransferases are required for palmitoylation-dependent regulation of large conductance calcium- and voltage-activated potassium channels. J Biol Chem.

[44] Li Y, Martin BR, Cravatt BF, Hofmann SL (2012). DHHC5 protein palmitoylates flotillin-2 and is rapidly degraded on induction of neuronal differentiation in cultured cells. J Biol Chem.

[45] Li Y (2010). DHHC5 interacts with PDZ domain 3 of post-synaptic density-95 (PSD-95) protein and plays a role in learning and memory. J Biol Chem.

[46] Yanai A (2006). Palmitoylation of huntingtin by HIP14 is essential for its trafficking and function. Nat Neurosci.

[47] Greaves J, Gorleku OA, Salaun C, Chamberlain LH (2010). Palmitoylation of the SNAP25 protein family: specificity and regulation by DHHC palmitoyl transferases. J Biol Chem.

[48] Milnerwood AJ (2013). Memory and synaptic deficits in Hip14/DHHC17 knockout mice. Proc Natl Acad Sci USA.

[49] Swarthout JT (2005). DHHC9 and GCP16 constitute a human protein fatty acyltransferase with specificity for H- and N-Ras. J Biol Chem.

[50] Percher A (2016). Mass-tag labeling reveals site-specific and endogenous levels of protein S-fatty acylation. Proc Natl Acad Sci USA.

[51] Mi H (2005). The PANTHER database of protein families, subfamilies, functions and pathways. Nucleic Acids Res.

[52] Wang J, Duncan D, Shi Z, Zhang B (2013). WEB-based GEne SeT AnaLysis Toolkit (WebGestalt): update 2013. Nucleic Acids Res.

