# Supplementary material for: Global, site-specific analysis of neuronal protein S-acylation
Source: Sci Rep. 2017 Jul 5;7:4683. doi: 10.1038/s41598-017-04580-1 (PMC5498535; doi:10.1038/s41598-017-04580-1)

Global, site-specific analysis of neuronal protein S-acylation.

Mark O. Collins, Keith T. Woodley & Jyoti S. Choudhary

Supplementary information

Figure S1: Palmitoylation sites identified in GPCRs

Figure S2. Fragmentation spectra of phosphorylated and previously palmitoylated peptides.

Figure S3. Uncropped image associated with Figure 6e

Figure S1: Palmitoylation sites identified in GPCRs

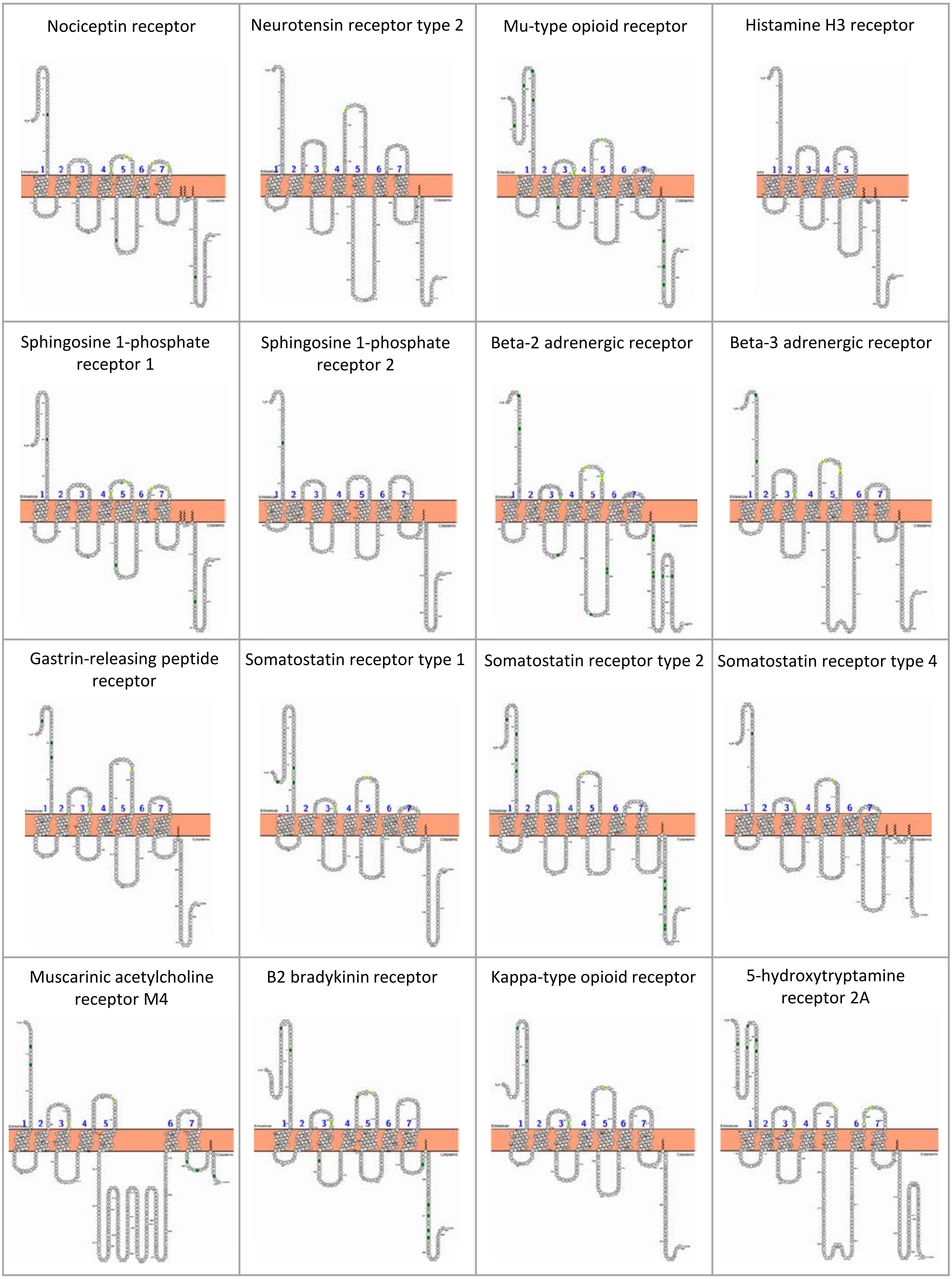

Cannabinoid receptor 1

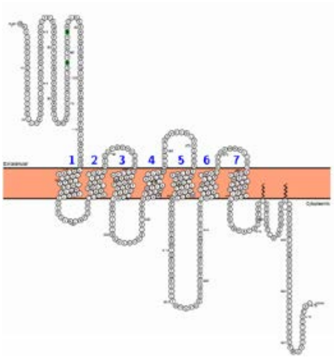

Endothelin B receptor

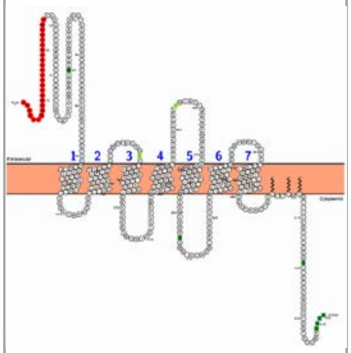

CX3C chemokine receptor 1

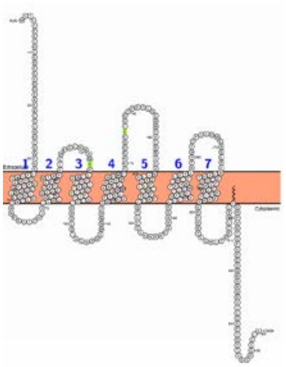

Lysophosphatidic acid receptor 1

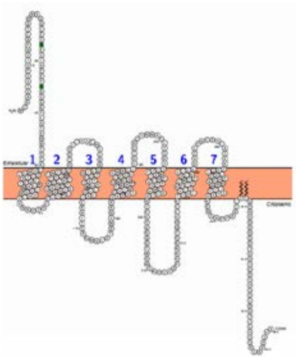

Neuropeptide Y receptor type 5

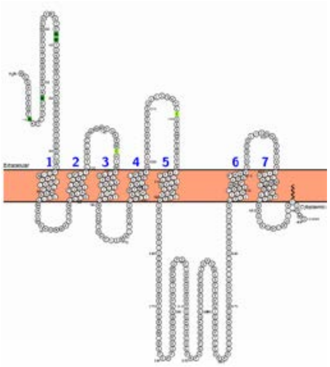

Adenosine receptor A2b

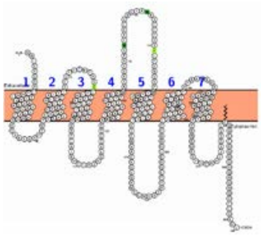

D(1A) dopamine receptor

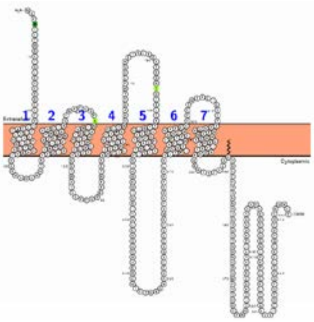

Uracil nucleotide/cysteinyl leukotriene receptor

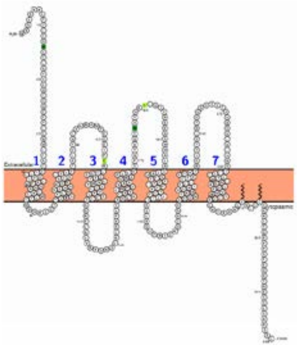

Probable G-protein coupled receptor 62

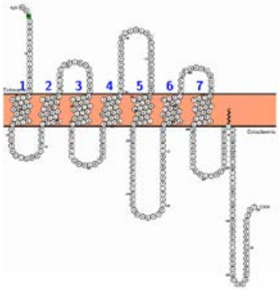

Neuropeptide FF receptor 2

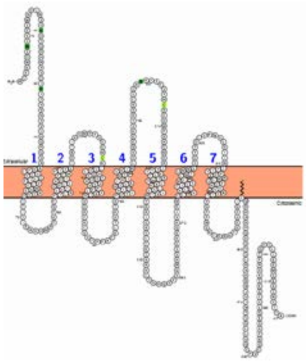

Endothelin B receptor-like protein 2

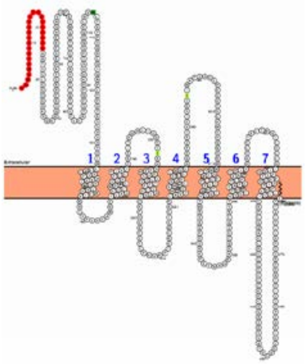

P2Y purinoceptor 12

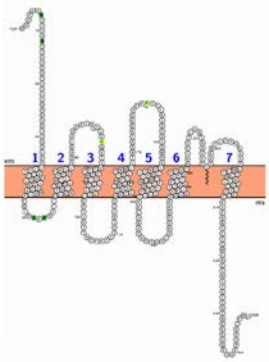

Muscarinic acetylcholine receptor M3

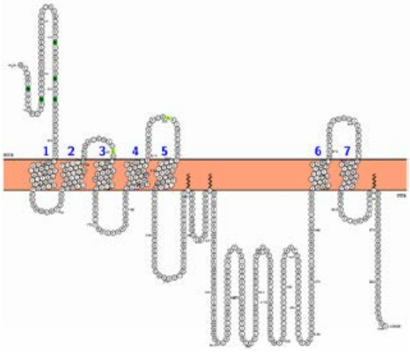

Muscarinic acetylcholine receptor M2

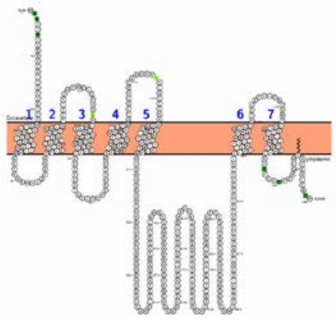

Prosaposin receptor GPR37

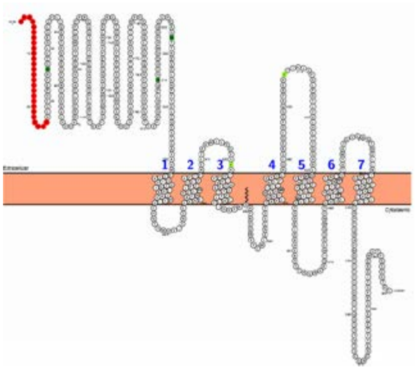

Probable G-protein coupled receptor 52

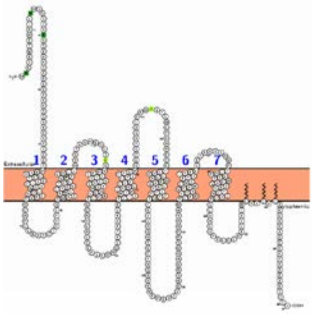

G-protein coupled receptor 161

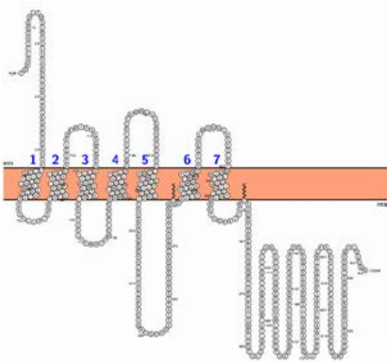

Probable G-protein coupled receptor 162

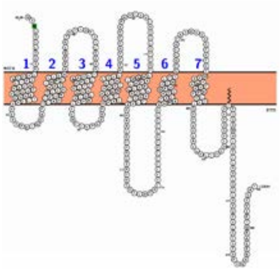

Latrophilin-3

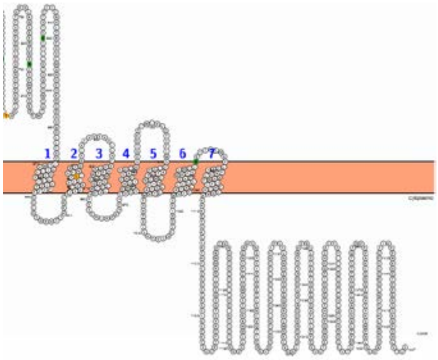

Latrophilin-2

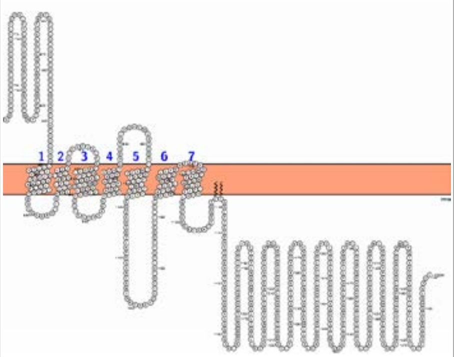

Vasoactive intestinal polypeptide receptor 2

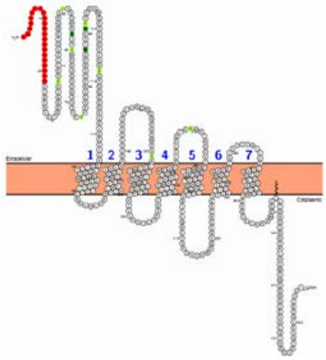

Pituitary adenylate cyclase-activating polypeptide type I receptor

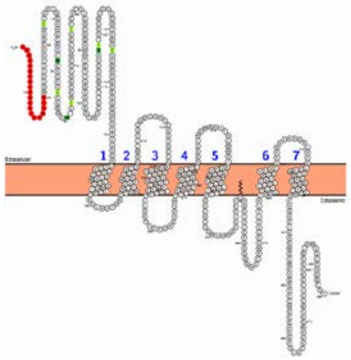

Frizzled-3

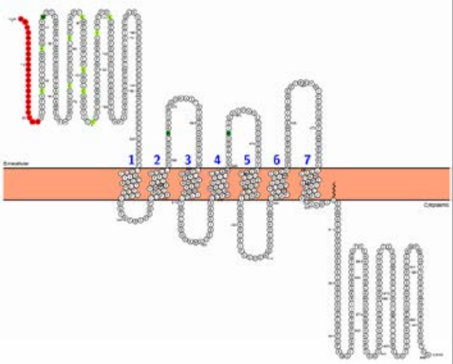

Probable G-protein coupled receptor 123

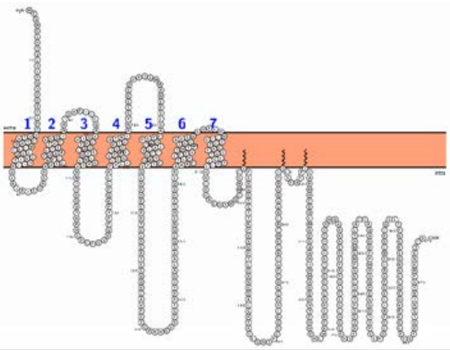

Figure S2. Fragmentation spectra of phosphorylated and previously palmitoylated peptides.

Raw file	Scan	Method	Score	m/z	Gene names
OTNCS_Brain_Palm_P2_2013July09-01	19562	ITMS; CID	279.1	929.93	Mpp2

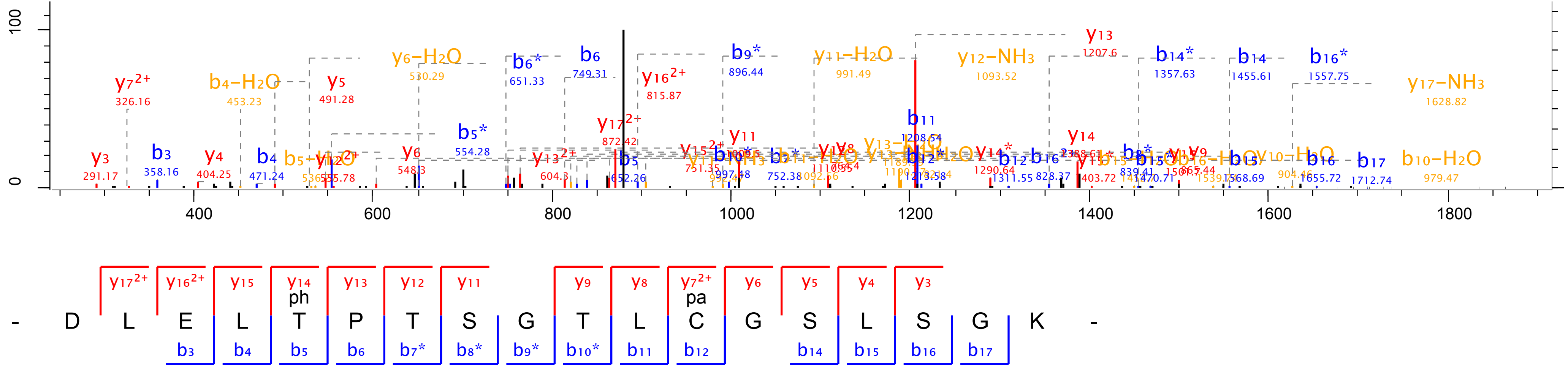

Raw file	Scan	Method	Score	m/z	Gene names
OTNCS_Brain_Palm_P2_2013July09-02	2560	ITMS; CID	141.36	1171.97	Rtn3

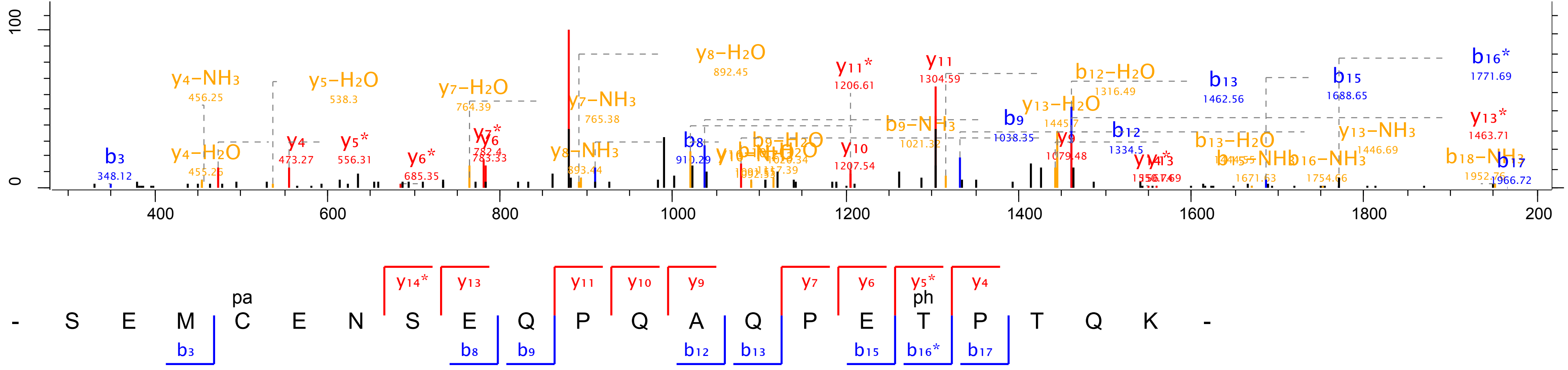

Raw file	Scan	Method	Score	m/z	Gene names
OTNCS_Brain_Palm_P2_2013July09-02	15576	ITMS; CID	128.36	550.75	Rgs9

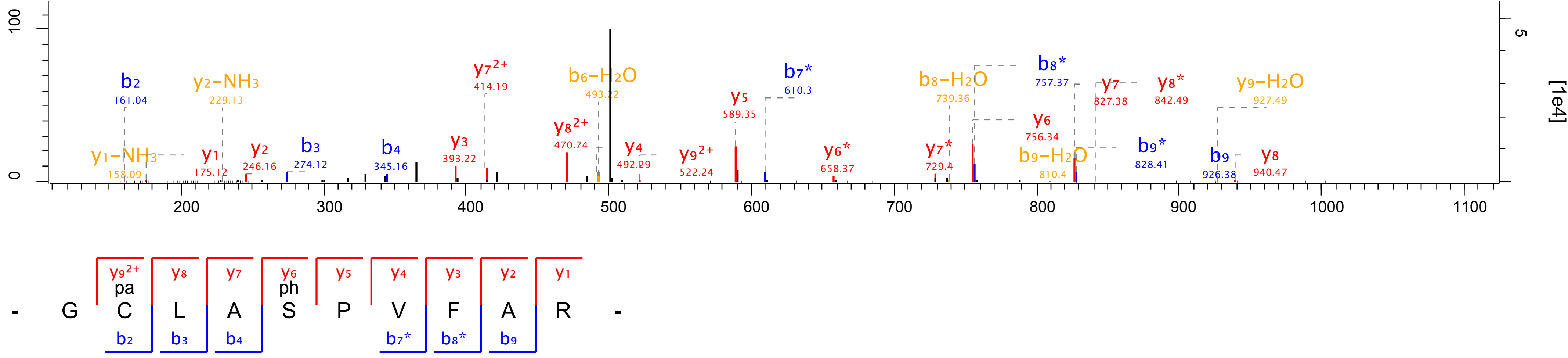

Raw file	Scan	Method	Score	m/z	Gene names
OTNCS_Brain_Palm_P2_2013July09-02	26689	ITMS; CID	57.77	1296.07	Lppr2

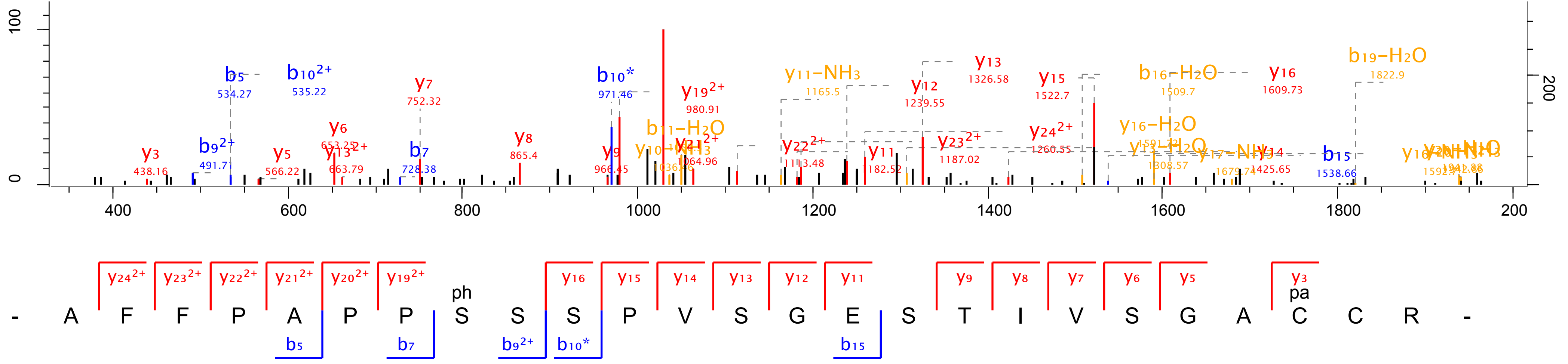

Raw file	Scan	Method	Score	m/z	Gene names
OTNCS_Brain_Palm_P2_2013July12-01	7434	ITMS; CID	41.16	711.59	Gpr123

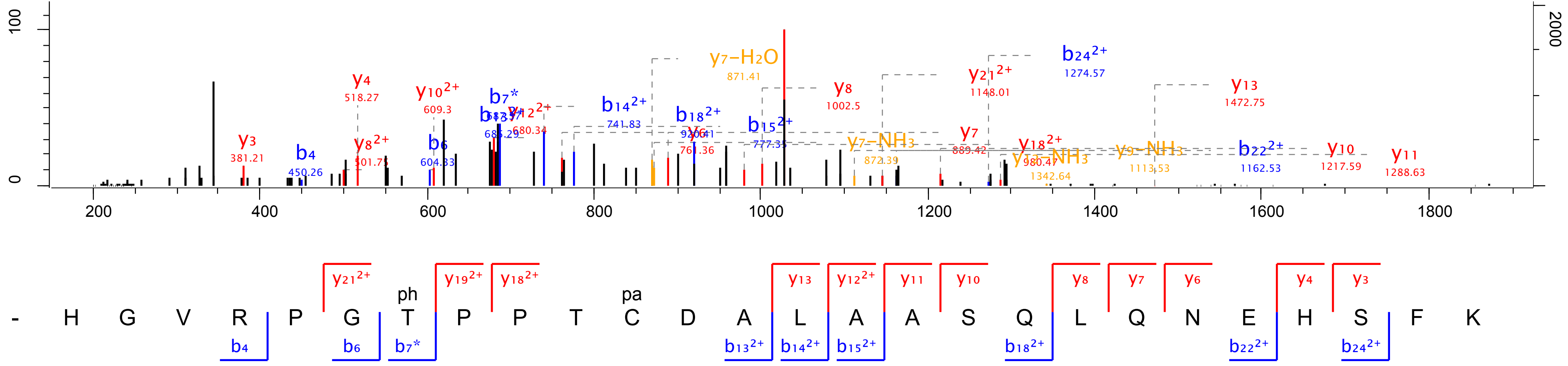

Raw file	Scan	Method	Score	m/z	Gene names
OTNCS_Brain_Palm_P2_2013July12-01	12266	ITMS; CID	90.9	1590.64	Cacna1c

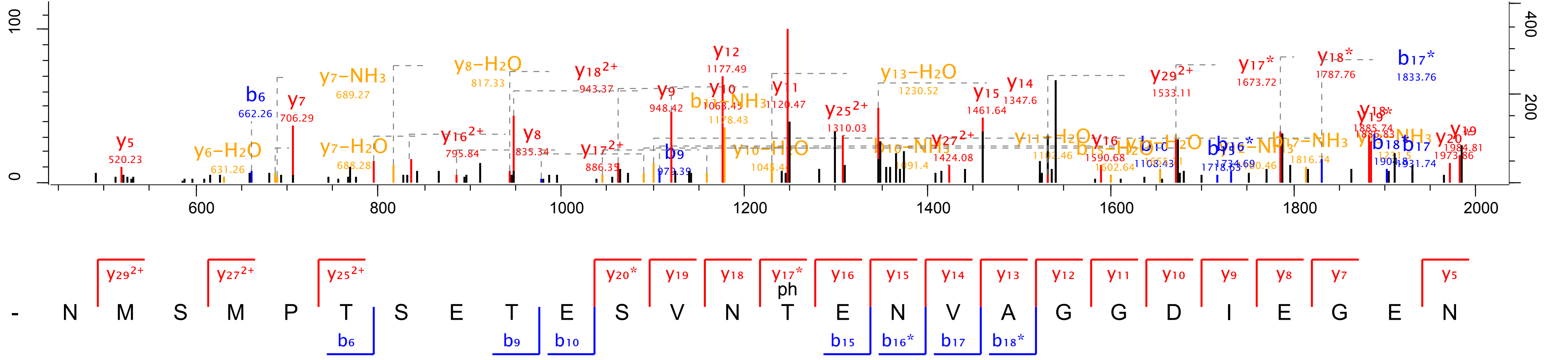

Raw file	Scan	Method	Score	m/z	Gene names
OTNCS_Brain_Palm_P2_2013July12-01	14478	ITMS; CID	158.83	1207.05	Ccny

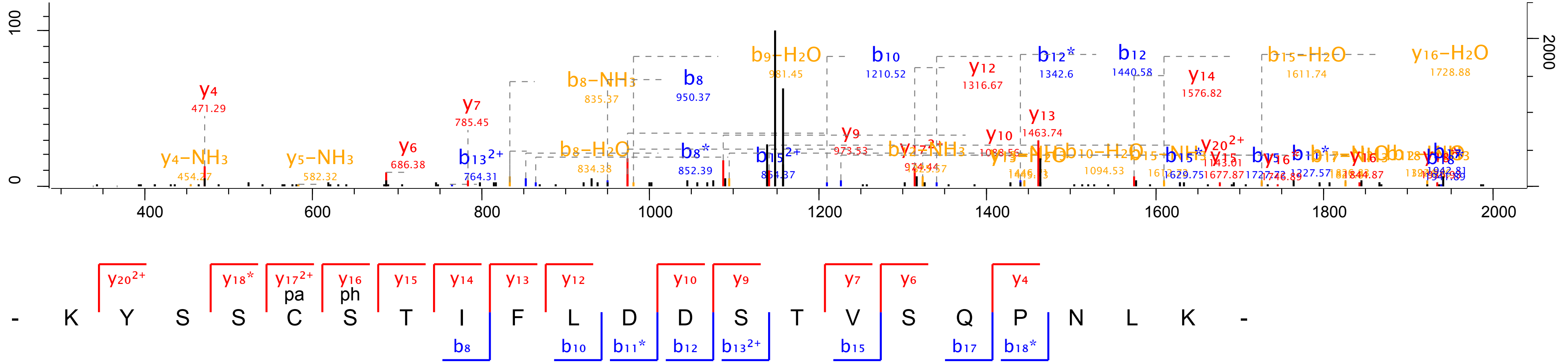

Raw file	Scan	Method	Score	m/z	Gene names
OTNCS_Brain_Palm_P2_2013July12-01	14701	ITMS; CID	49.45	976.94	Grin2b

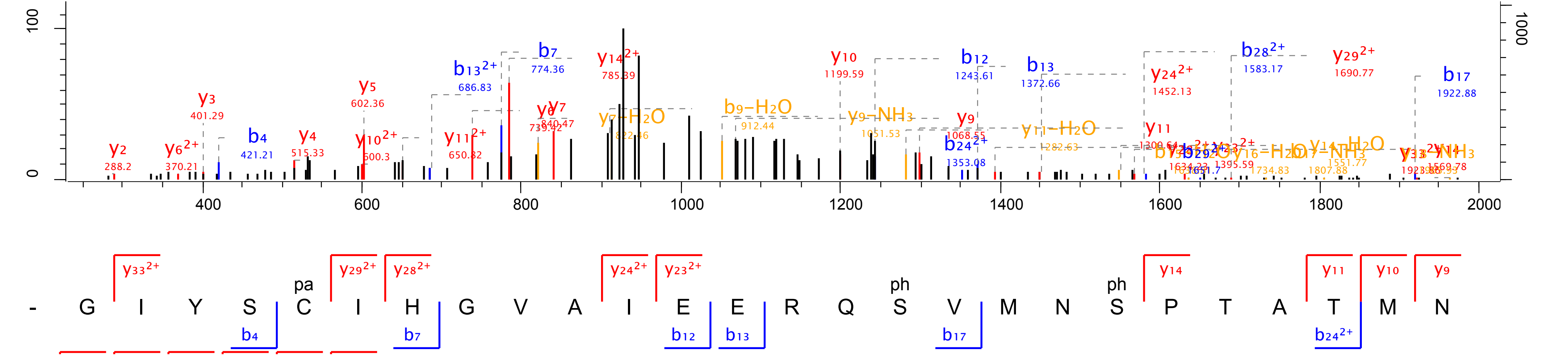

Raw file	Scan	Method	Score	m/z	Gene names
OTNCS_Brain_Palm_P2_2013July12-01	15924	ITMS; CID	89.5	946.92	Snrk

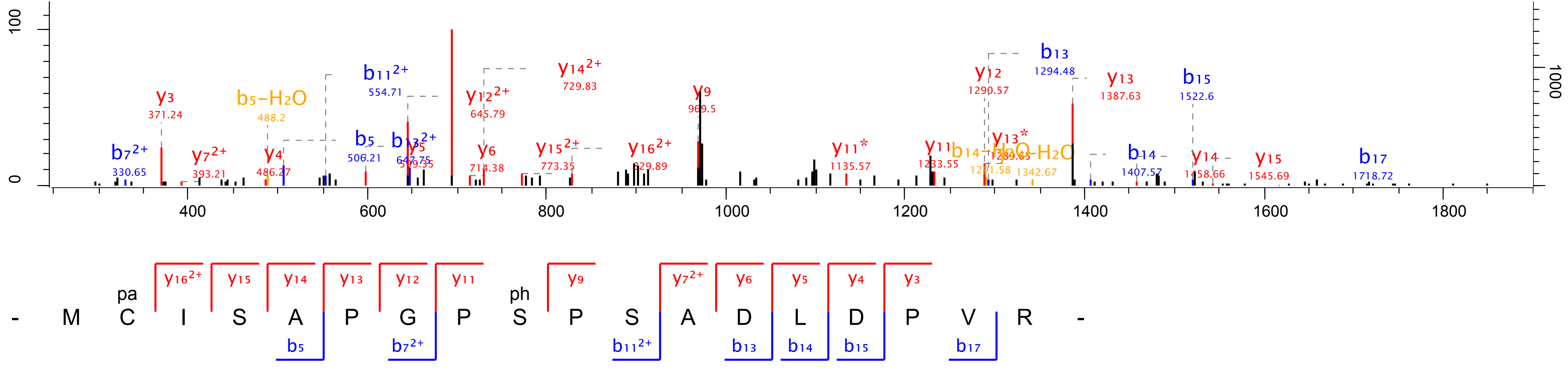

Raw file	Scan	Method	Score	m/z	Gene names
OTNCS_Brain_Palm_P2_2013July12-01	18985	ITMS; CID	125.37	1296.57	Lppr2

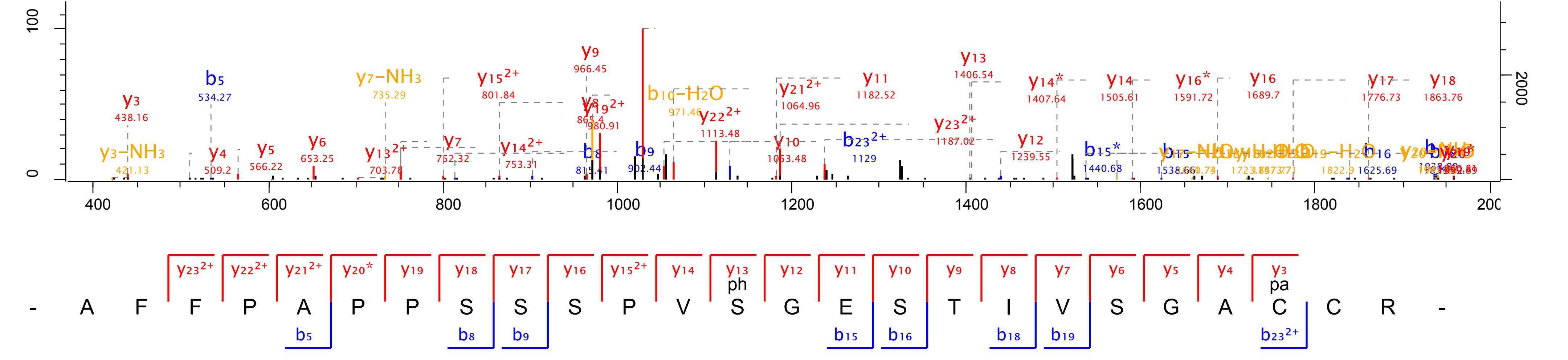

Raw file	Scan	Method	Score	m/z	Gene names
OTNCS_Brain_Palm_P2_2013July12-02	13849	ITMS; CID	91.87	1404.04	Cnr1

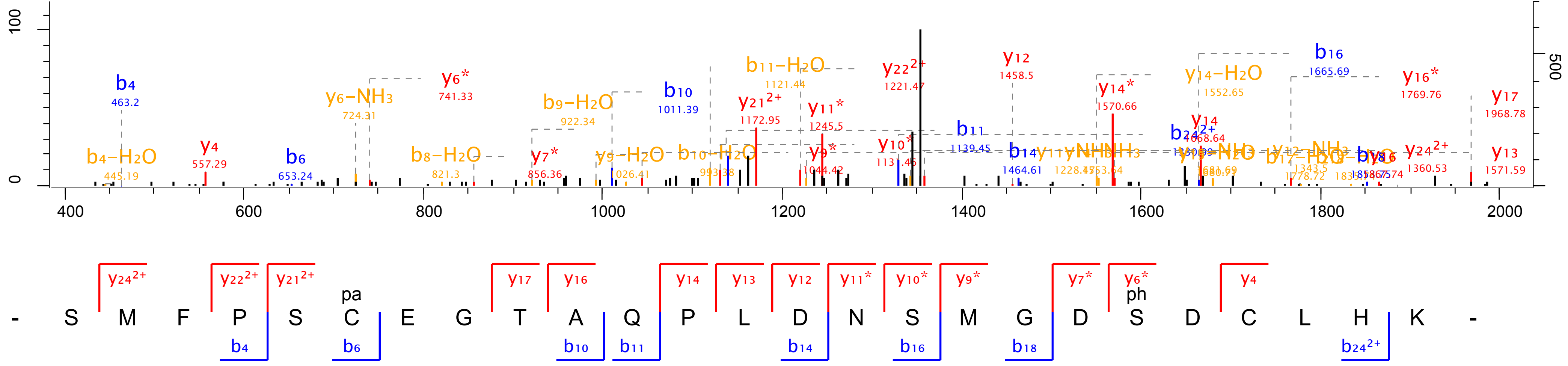

Raw file	Scan	Method	Score	m/z	Gene names
OTNCS_Brain_Palm_P2_2013July12-02	13899	ITMS; CID	151.28	973.42	Rgs8

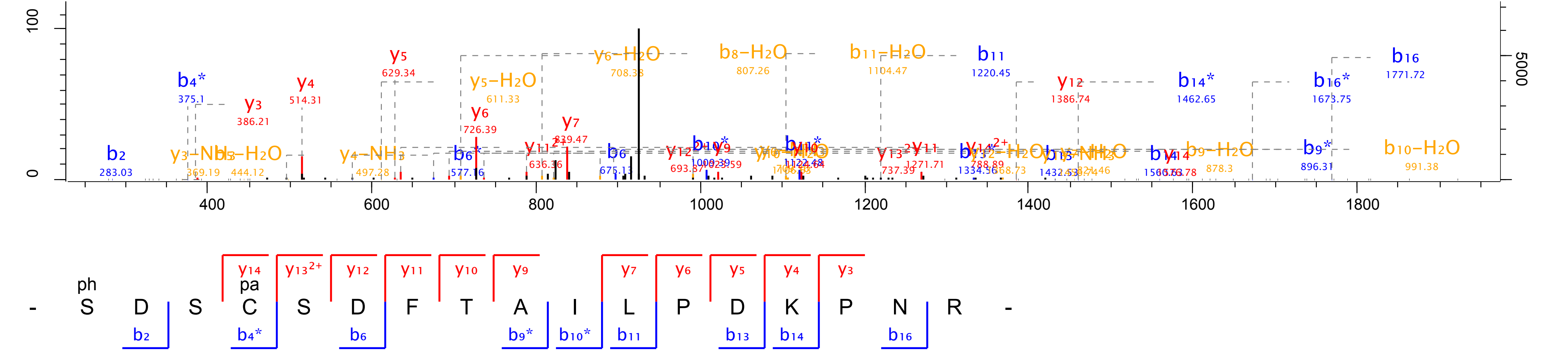

Raw file	Scan	Method	Score	m/z	Gene names
OTNCS_Brain_Palm_P2_2013July12-02	14390	ITMS; CID	106.36	1207.56	Ccny

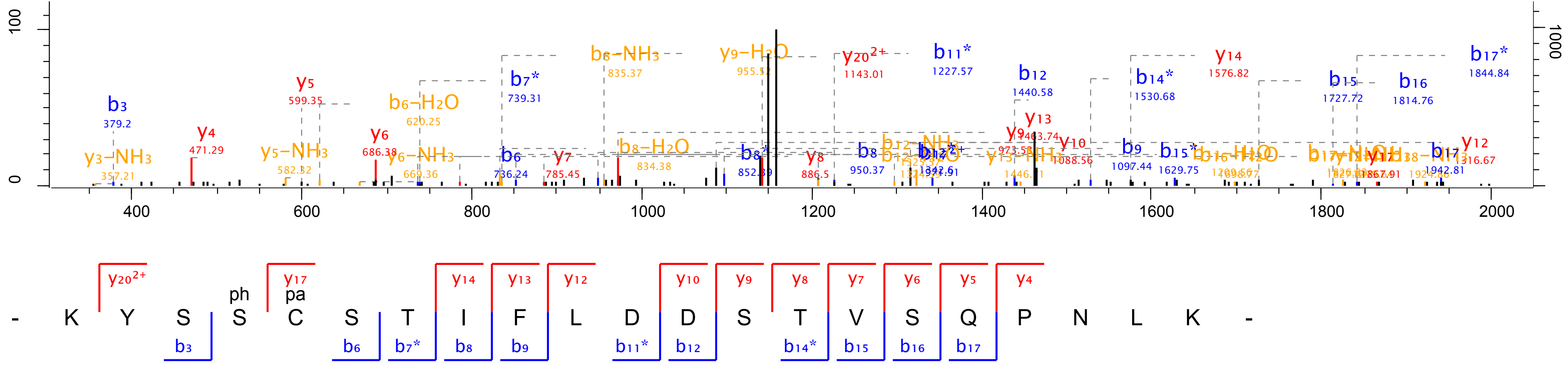

Raw file	Scan	Method	Score	m/z	Gene names
OTNCS_Brain_Palm_P2_2013July12-02	19321	ITMS; CID	73.67	1296.07	Lppr2

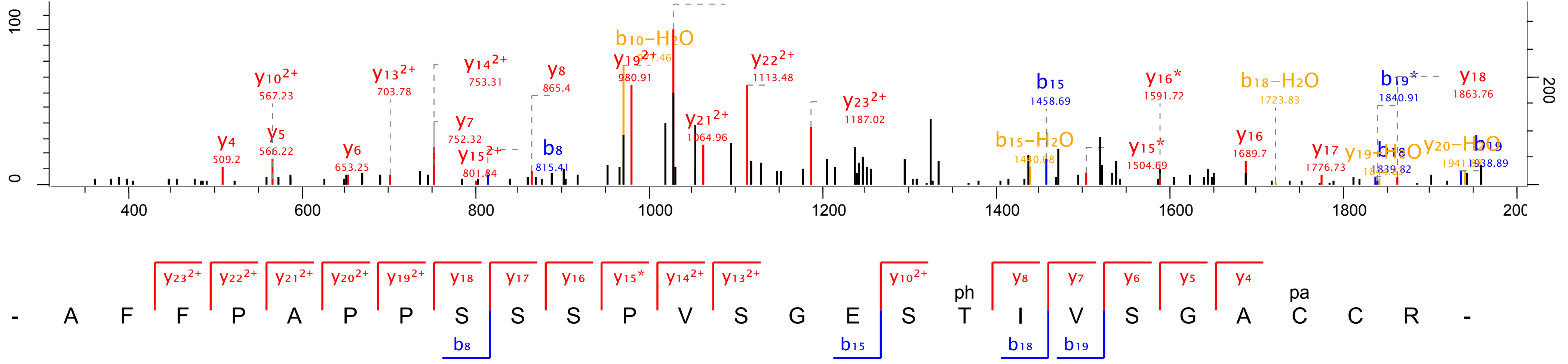

Raw file	Scan	Method	Score	m/z	Gene names
OTNCS_Brain_Palm_P4_2013July09-01	13756	ITMS; CID	88.77	936.7	Cnr1

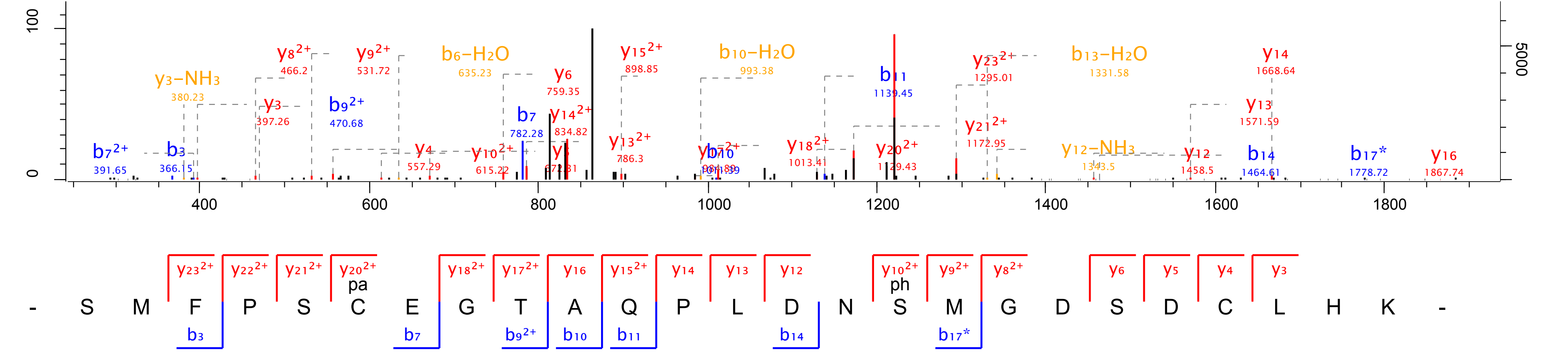

Raw file	Scan	Method	Score	m/z	Gene names
OTNCS_Brain_Palm_P4_2013July09-01	14490	ITMS; CID	235.61	995.93	Oprm1

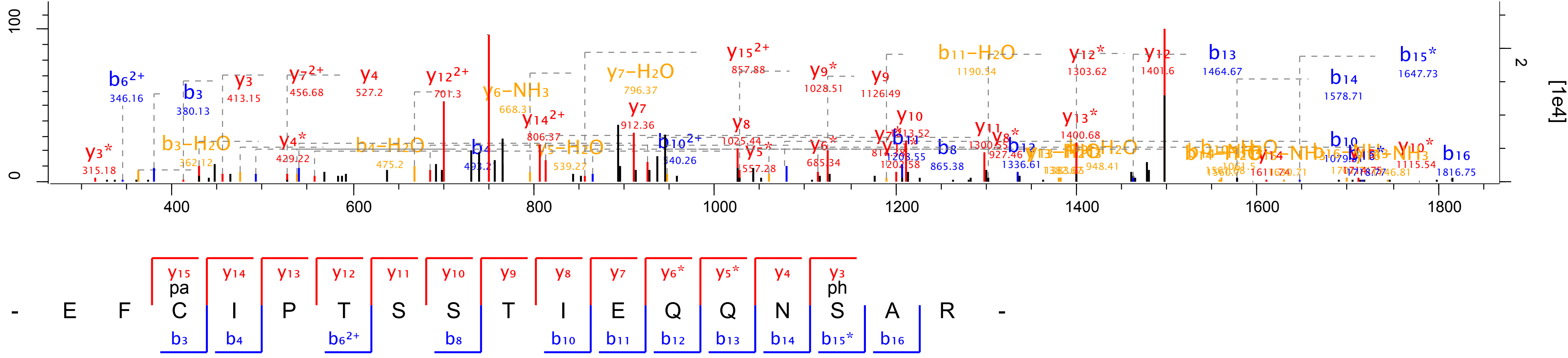

Raw file	Scan	Method	Score	m/z	Gene names
OTNCS_Brain_Palm_P4_2013July09-01	18675	ITMS; CID	85.18	1086.99	Atp1b2

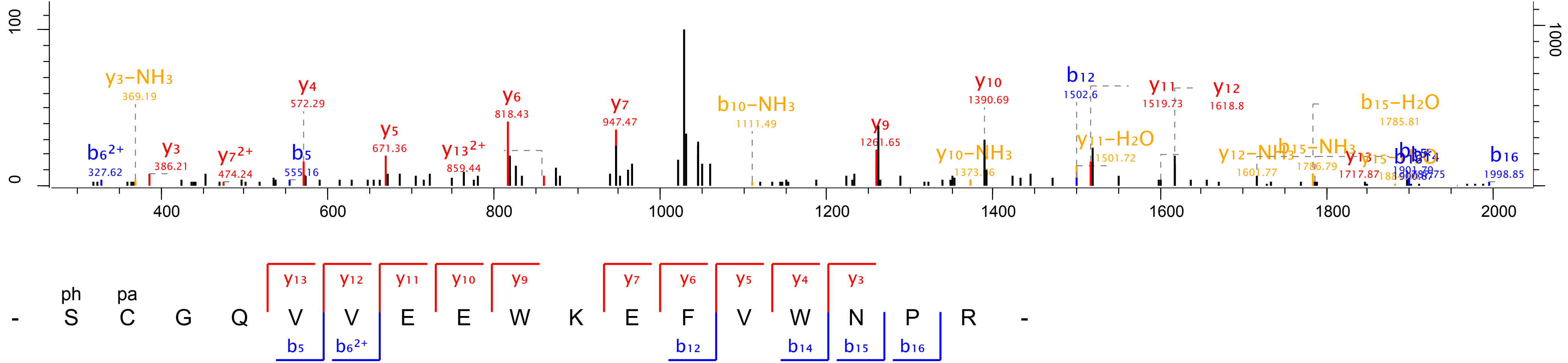

Raw file	Scan	Method	Score	m/z	Gene names
OTNCS_Brain_Palm_P4_2013July09-01	18959	ITMS; CID	84.37	1296.57	Lppr2

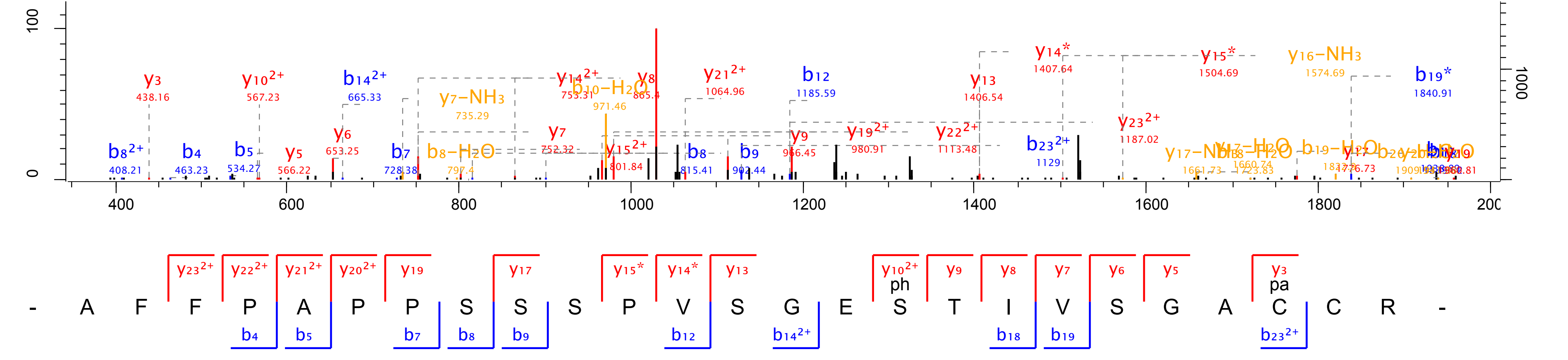

Raw file	Scan	Method	Score	m/z	Gene names
OTNCS_Brain_Palm_P4_2013July09-01	21179	ITMS; CID	48.35	994.17	Daam2

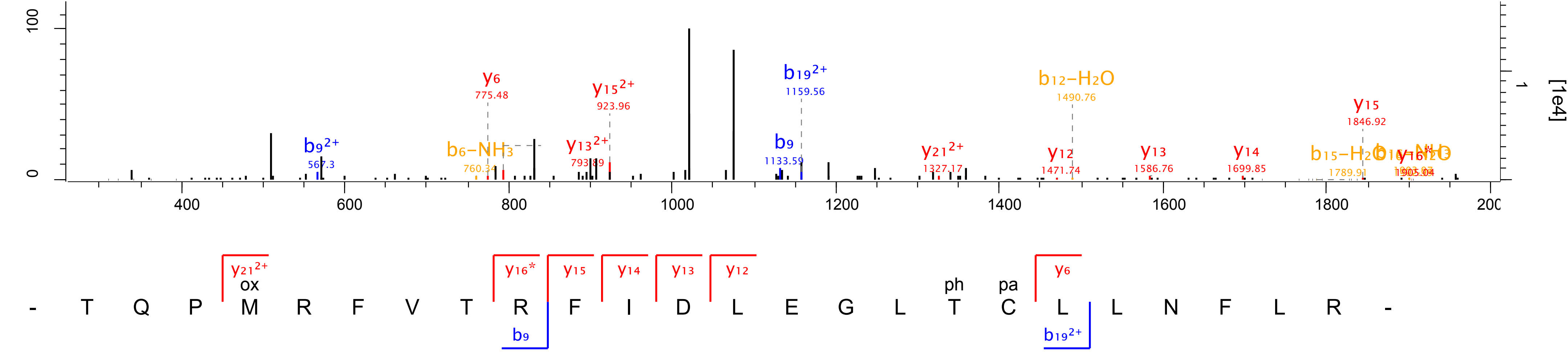

Raw file	Scan	Method	Score	m/z	Gene names
OTNCS_Brain_Palm_P4_2013July09-01	22396	ITMS; CID	65.06	1276.59	Slc39a14

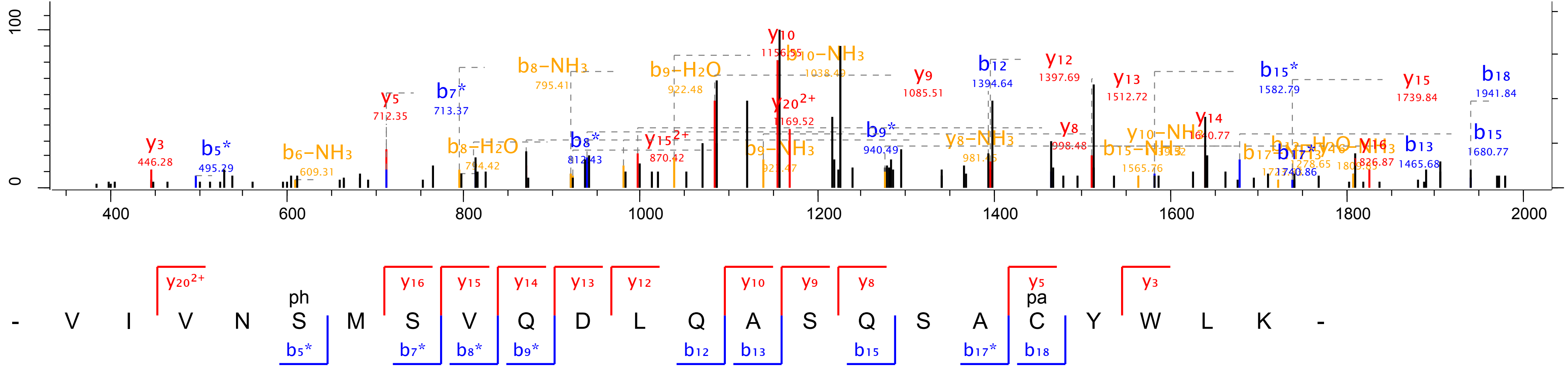

Raw file	Scan	Method	Score	m/z	Gene names
OTNCS_Brain_Palm_P2_2013July09-01	5086	ITMS; CID	130.51	1007.91	Prr24

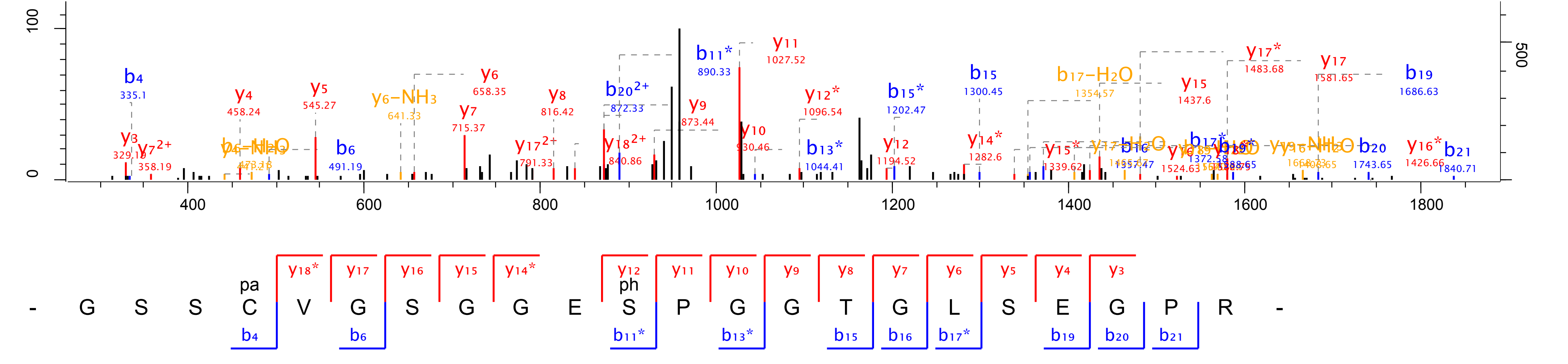

Raw file	Scan	Method	Score	m/z	Gene names
OTNCS_Brain_Palm_P2_2013July09-01	14470	ITMS; CID	129.62	1179.51	Csnk1g3

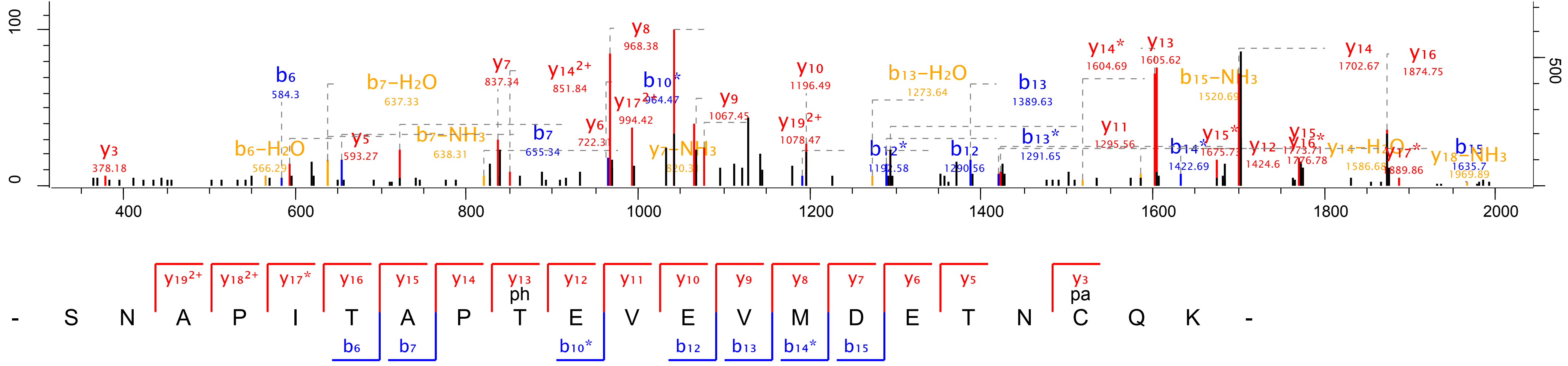

Raw file	Scan	Method	Score	m/z	Gene names
OTNCS_Brain_Palm_P2_2013July09-01	14598	ITMS; CID	47.18	977.44	Grin2b

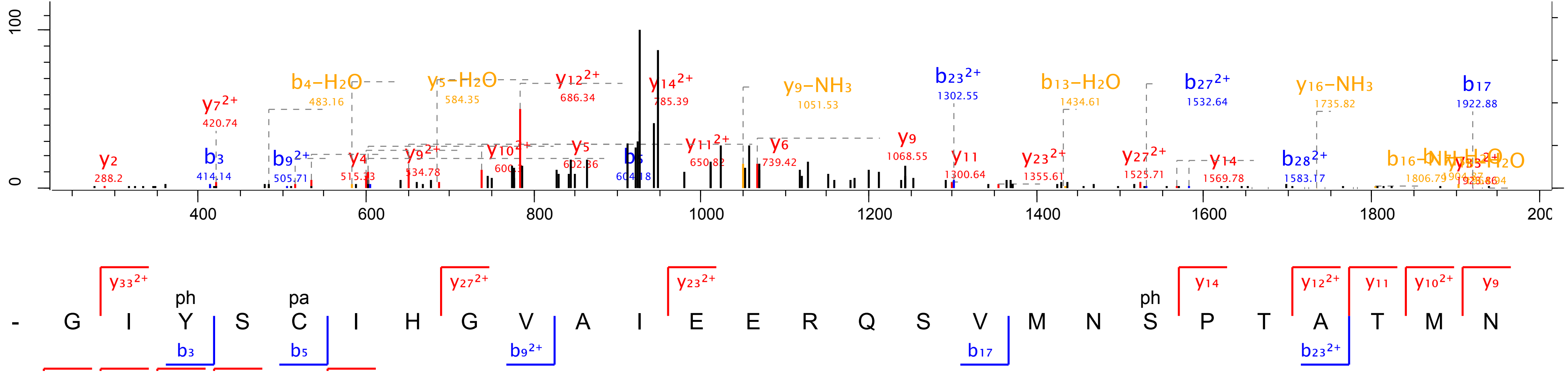

Raw file	Scan	Method	Score	m/z	Gene names
OTNCS_Brain_Palm_P4_2013July09-02	27491	ITMS; CID	66.37	1296.57	Lppr2

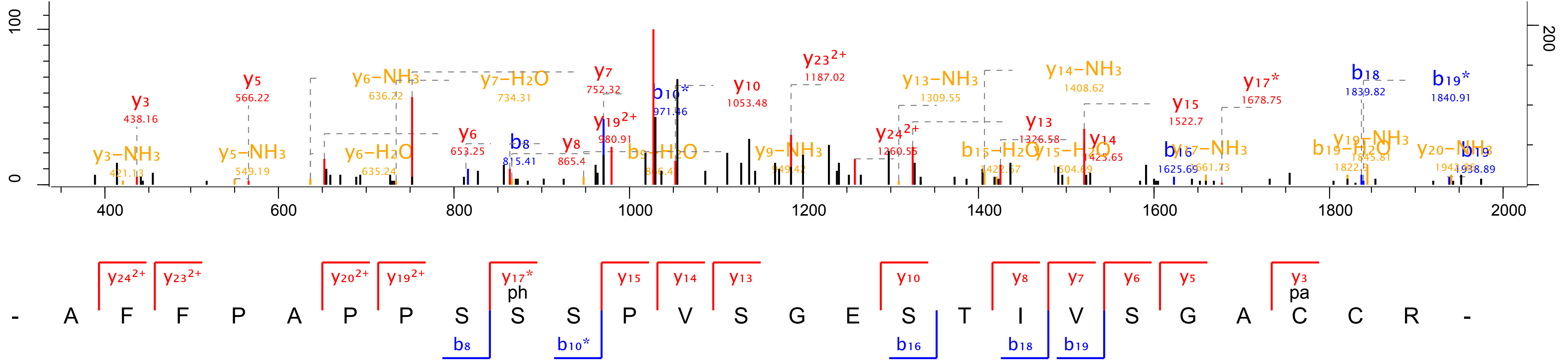

Raw file	Scan	Method	Score	m/z	Gene names
OTNCS_Brain_Palm_P4_2013July12-01	7807	ITMS; CID	68.41	751.09	Ccny

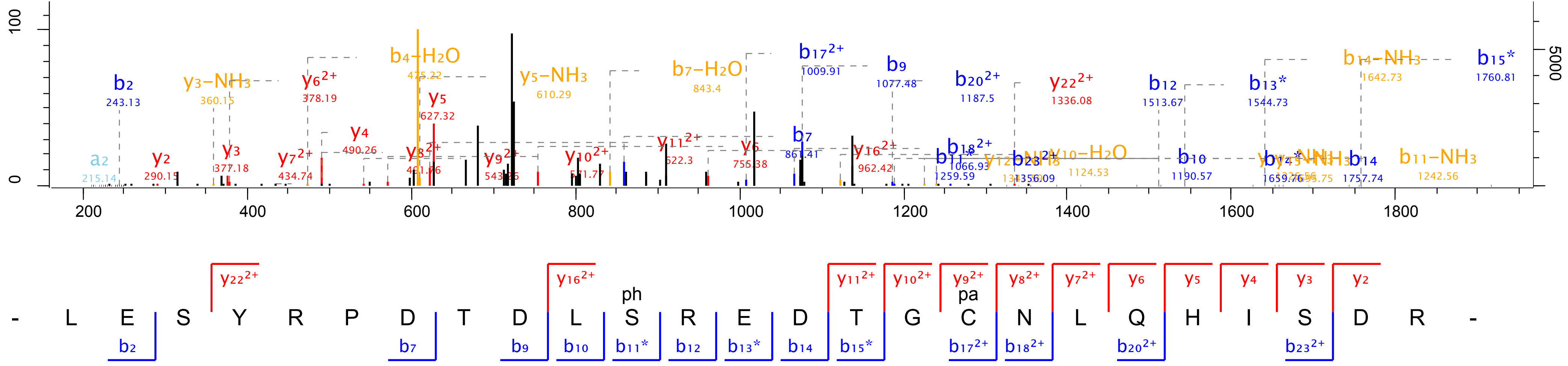

Raw file	Scan	Method	Score	m/z	Gene names
OTNCS_Brain_Palm_P4_2013July12-01	18667	ITMS; CID	73.77	1467.13	Hrh3

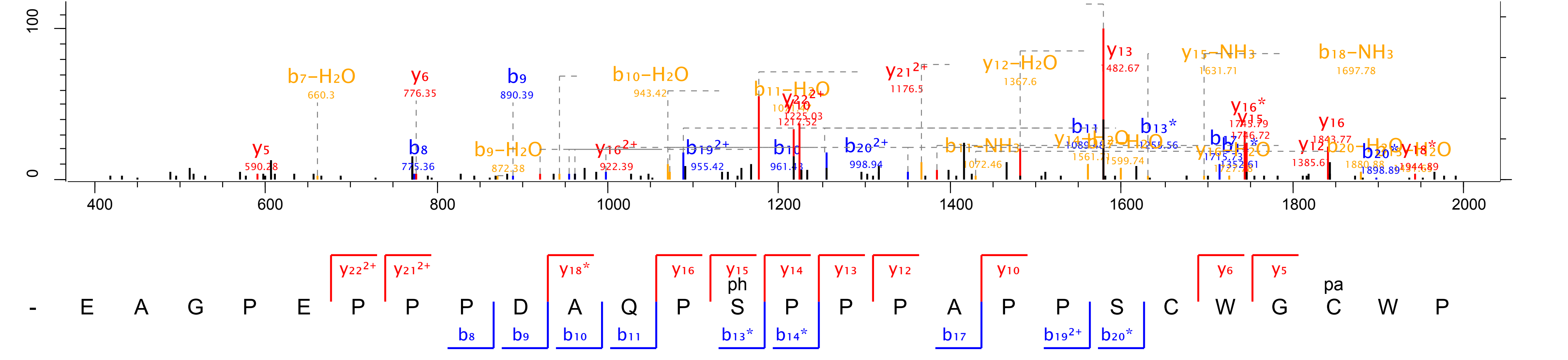

Raw file	Scan	Method	Score	m/z	Gene names
OTNCS_Brain_Palm_P4_2013July12-01	20750	ITMS; CID	75.87	1228.07	Lrrc7

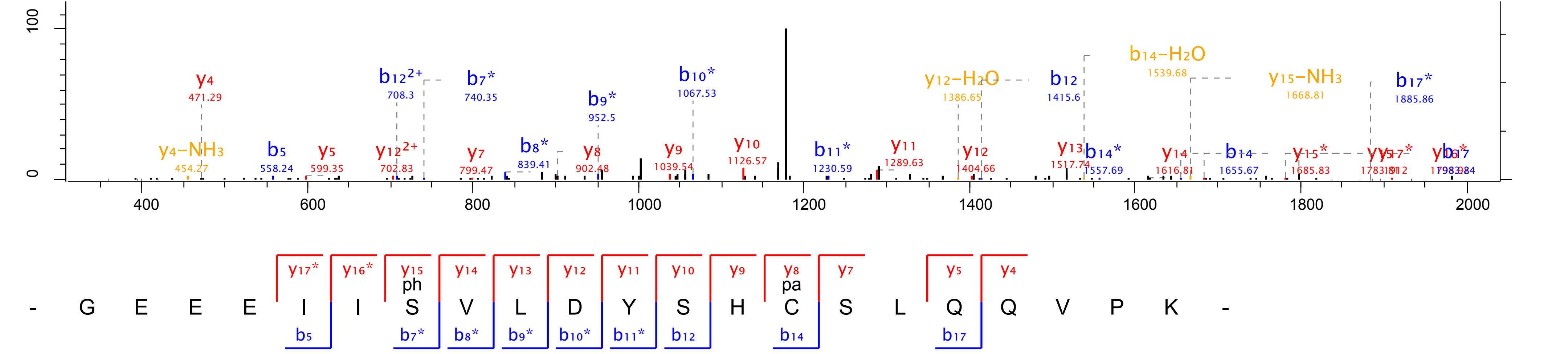

Raw file	Scan	Method	Score	m/z	Gene names
OTNCS_Brain_Palm_P4_2013July12-01	20860	ITMS; CID	78.27	1091.95	Cacng7

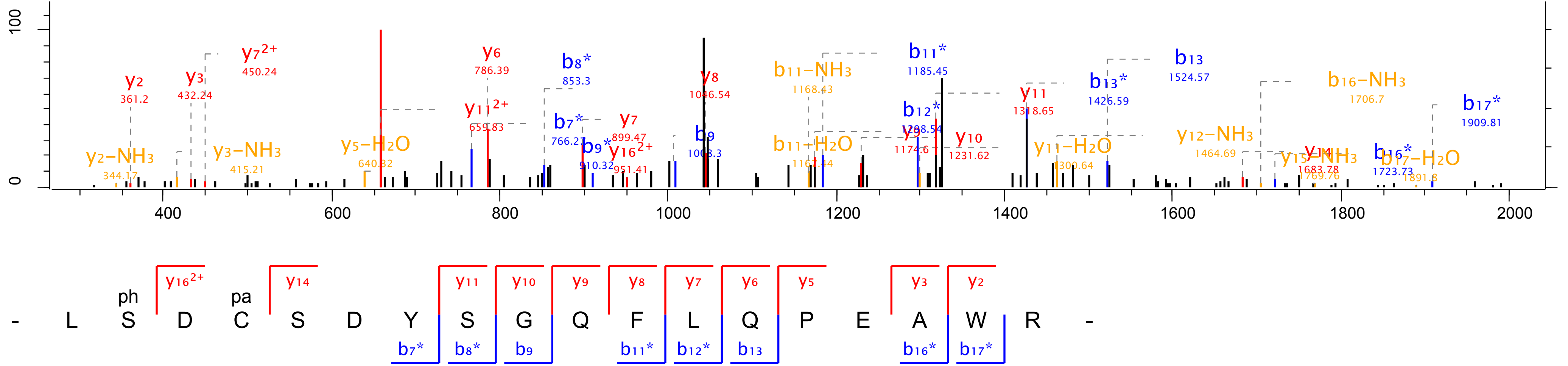

Raw file	Scan	Method	Score	m/z	Gene names
OTNCS_Brain_Palm_P4_2013July12-01	23765	ITMS; CID	60.32	707.33	Pebp1

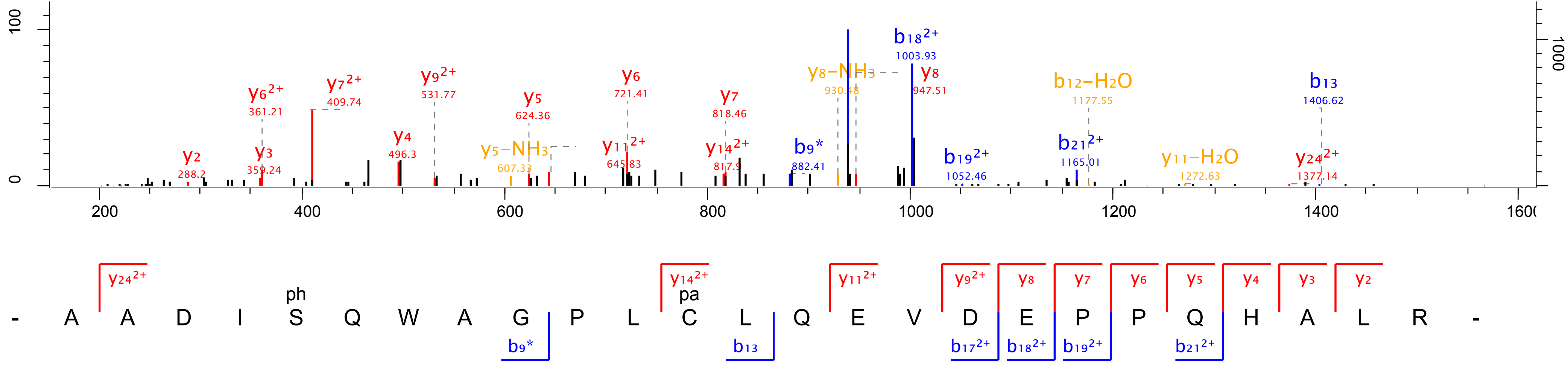

Raw file	Scan	Method	Score	m/z	Gene names
OTNCS_Brain_Palm_P4_2013July12-01	24223	ITMS; CID	70.57	1317.07	Cacna1h

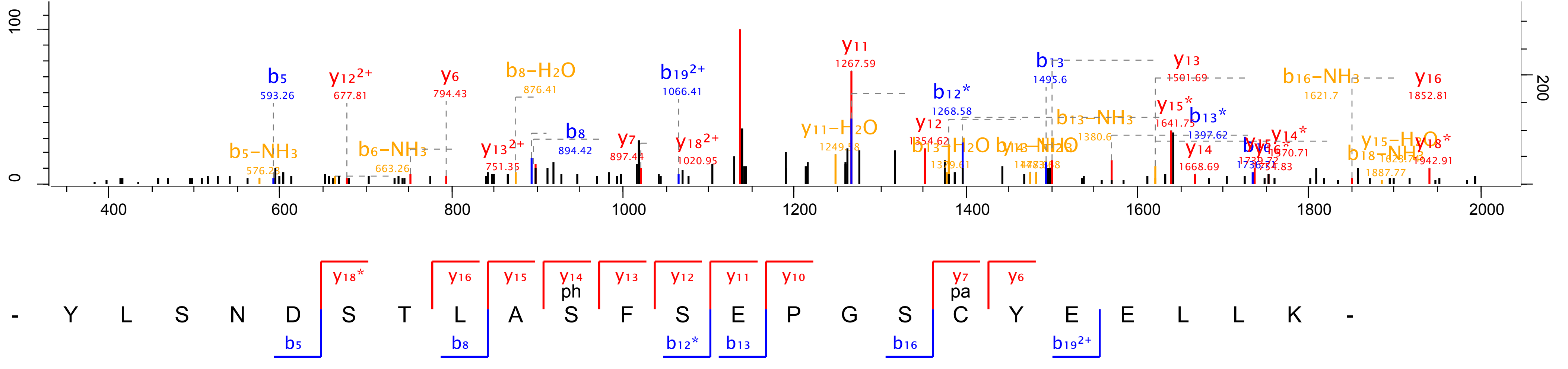

Raw file	Scan	Method	Score	m/z	Gene names
OTNCS_Brain_Palm_P4_2013July12-02	7850	ITMS; CID	52.27	751.08	Ccny

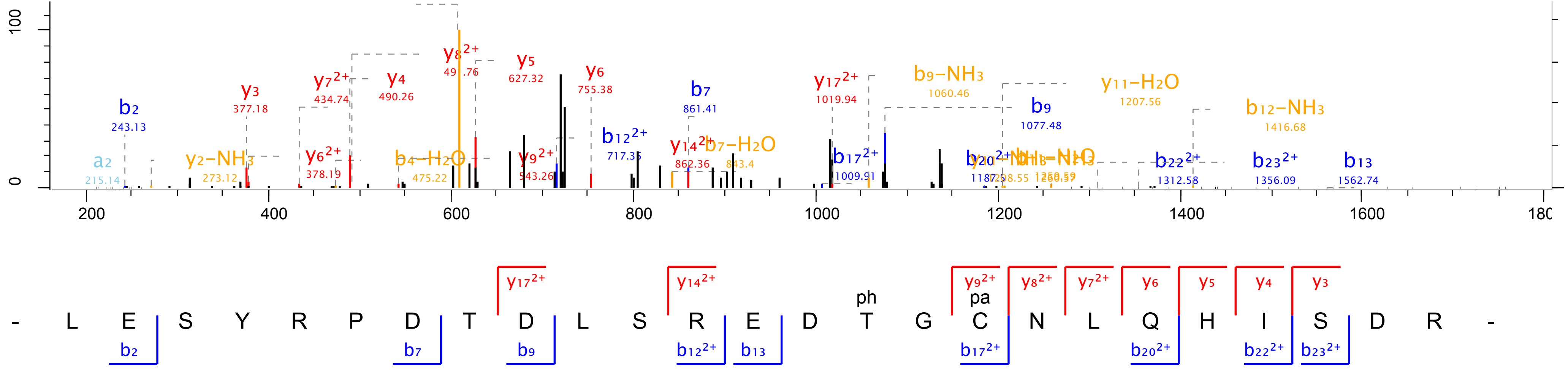

Raw file	Scan	Method	Score	m/z	Gene names
OTNCS_Brain_Palm_P4_2013July12-02	13899	ITMS; CID	131.9	973.42	Rgs8

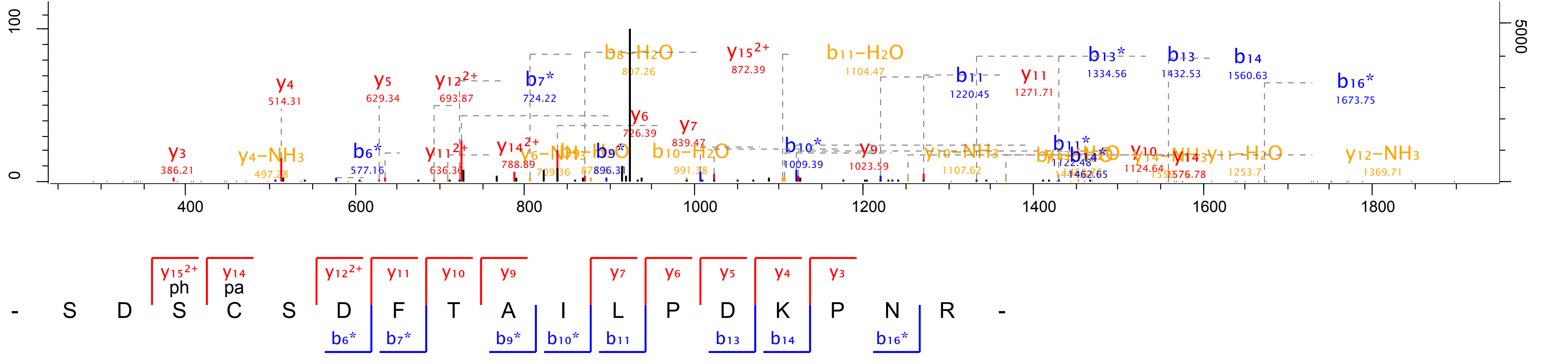

Raw file	Scan	Method	Score	m/z	Gene names
OTNCS_Brain_Palm_P4_2013July12-02	18772	ITMS; CID	98.15	1296.57	Lppr2

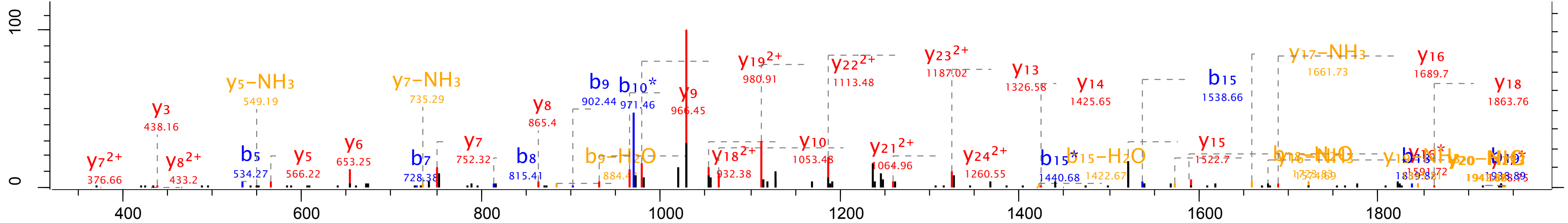

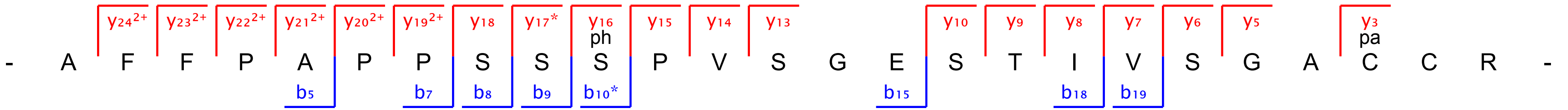

Raw file	Scan	Method	Score	m/z	Gene names
OTNCS_Brainpeppalm_2012Dec12-P1-01	4987	ITMS; CID	105.99	983.92	Kcnk1

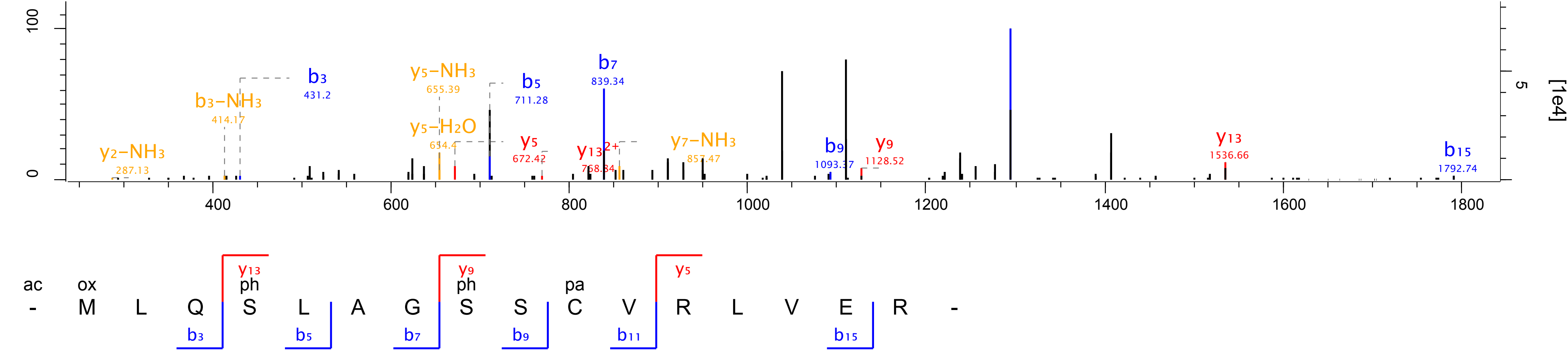

Raw file	Scan	Method	Score	m/z	Gene names
OTNCS_Brainpeppalm_2012Dec12-P1-01	9482	ITMS; CID	105.14	571.76	Xkr4

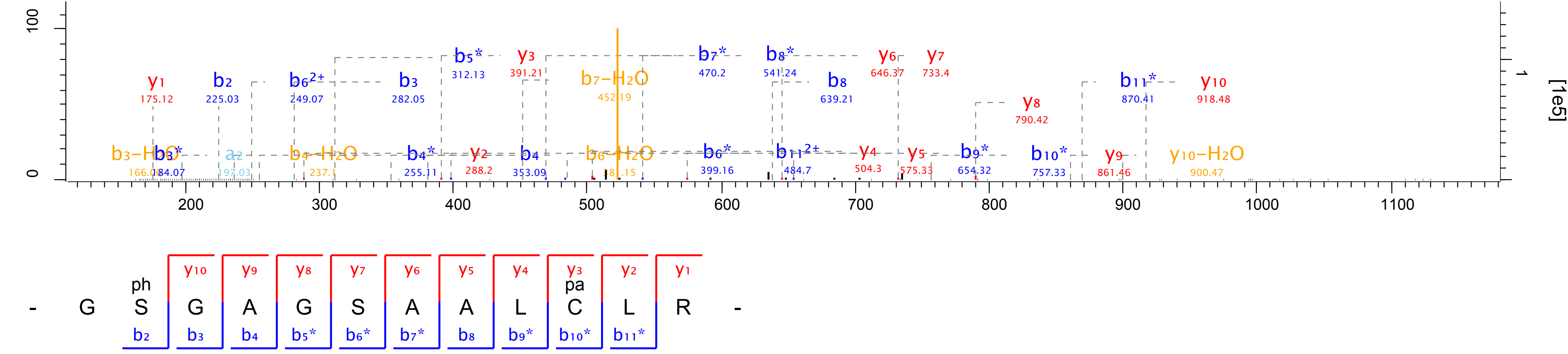

Raw file	Scan	Method	Score	m/z	Gene names
OTNCS_Brainpeppalm_2012Dec12-P1-02	11772	ITMS; CID	106.58	970.44	Dtnb

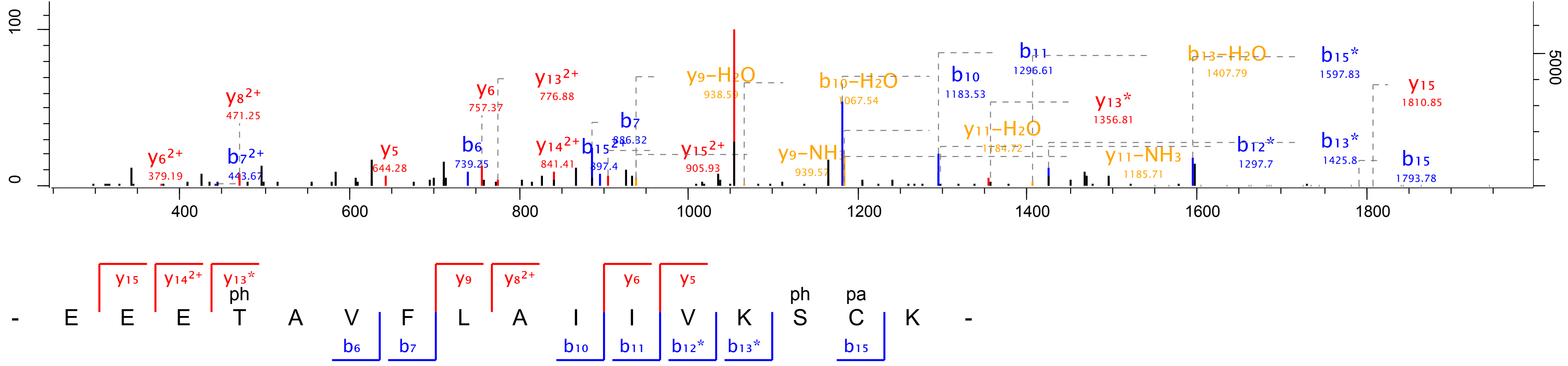

Raw file	Scan	Method	Score	m/z	Gene names
OTNCS_Brainpeppalm_2012Dec12-P1-02	16586	ITMS; CID	54.99	1078.82	Drd1

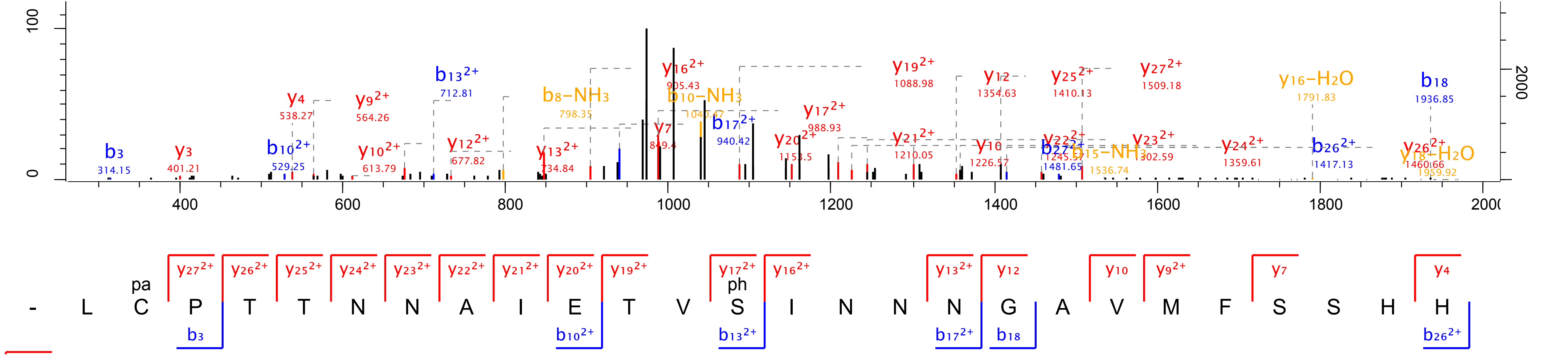

Raw file	Scan	Method	Score	m/z	Gene names
OTNCS_Brainpeppalm_2012Dec12-P1-02	18722	ITMS; CID	76.91	1303.22	Ctnnd2

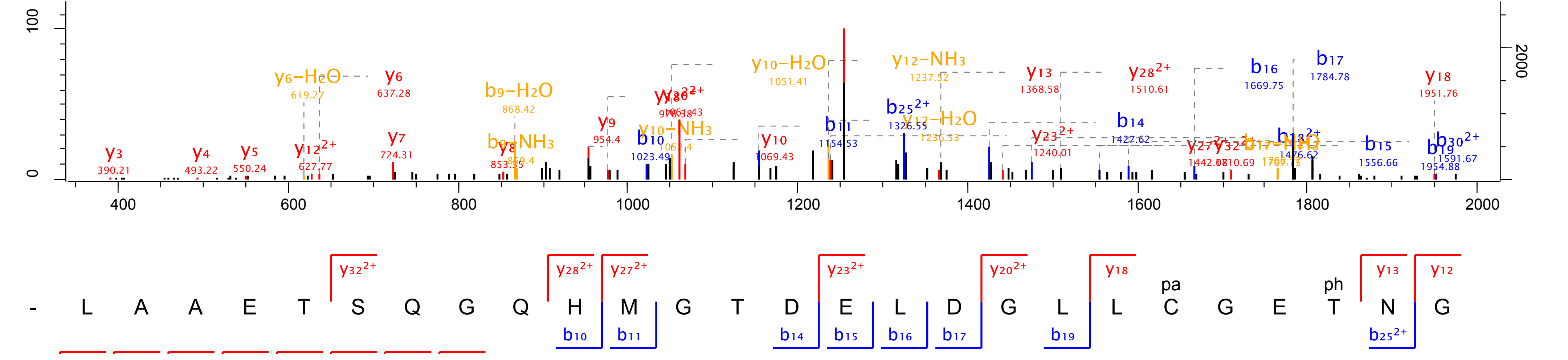

Raw file	Scan	Method	Score	m/z	Gene names
OTNCS_Brainpeppalm_2012Dec12-P1-02	18773	ITMS; CID	93.55	1010.41	Sept5

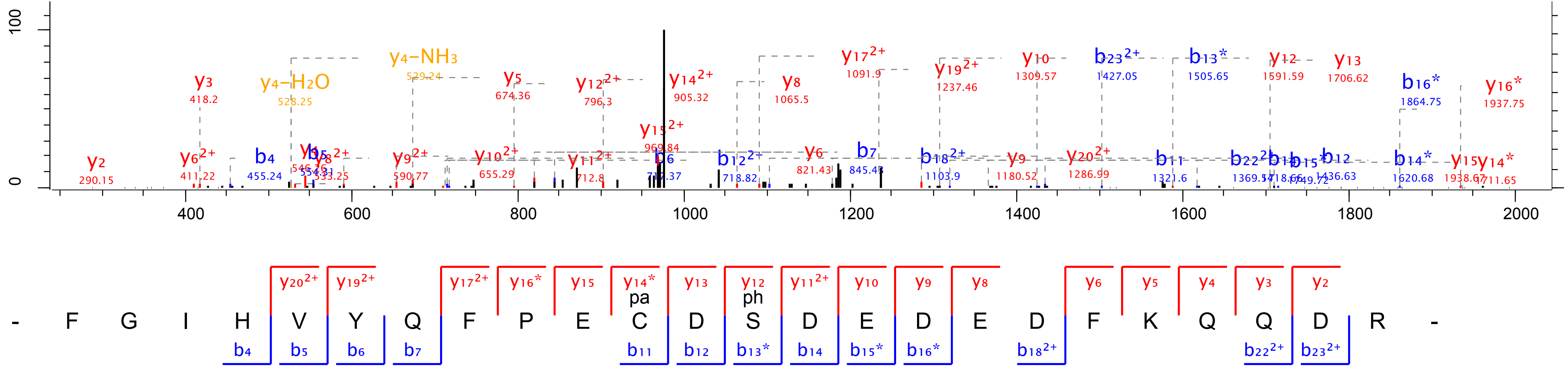

Raw file	Scan	Method	Score	m/z	Gene names
OTNCS_Brainpeppalm_2012Dec12-P1-02	22431	ITMS; CID	84.4	929.92	Mpp2

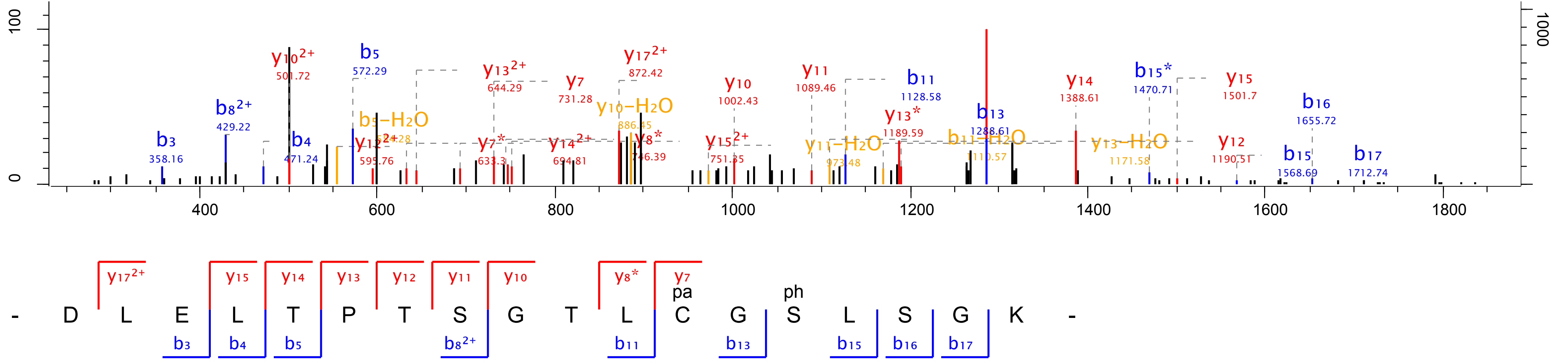

Raw file	Scan	Method	Score	m/z	Gene names
OTNCS_Brainpeppalm_2012Dec12-P1-02	23347	ITMS; CID	212.24	929.93	Mpp2

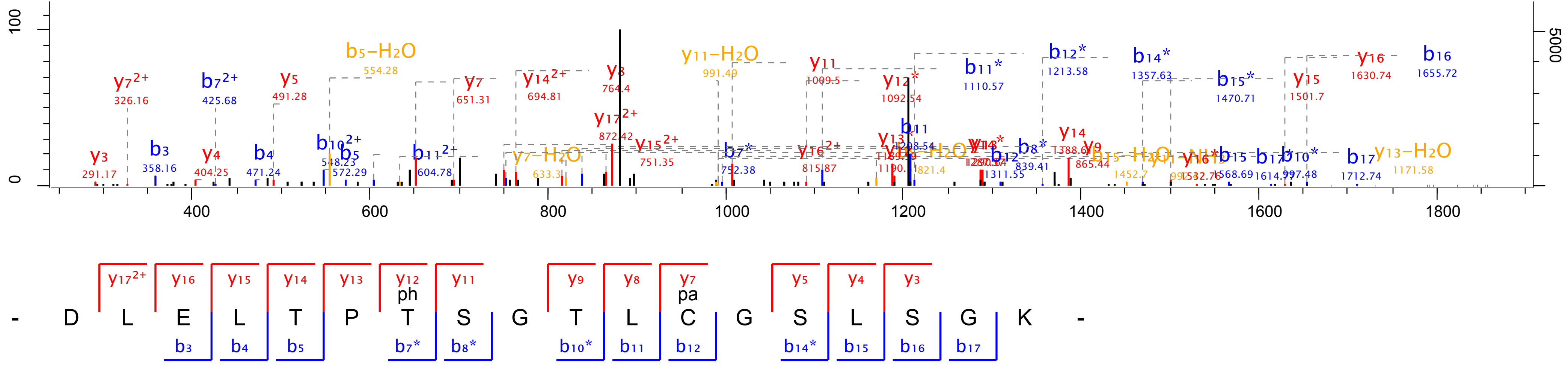

Raw file	Scan	Method	Score	m/z	Gene names
OTNCS_Brainpeppalm_2012Nov30-P1-01	16514	ITMS; CID	90.56	930.43	Mpp2

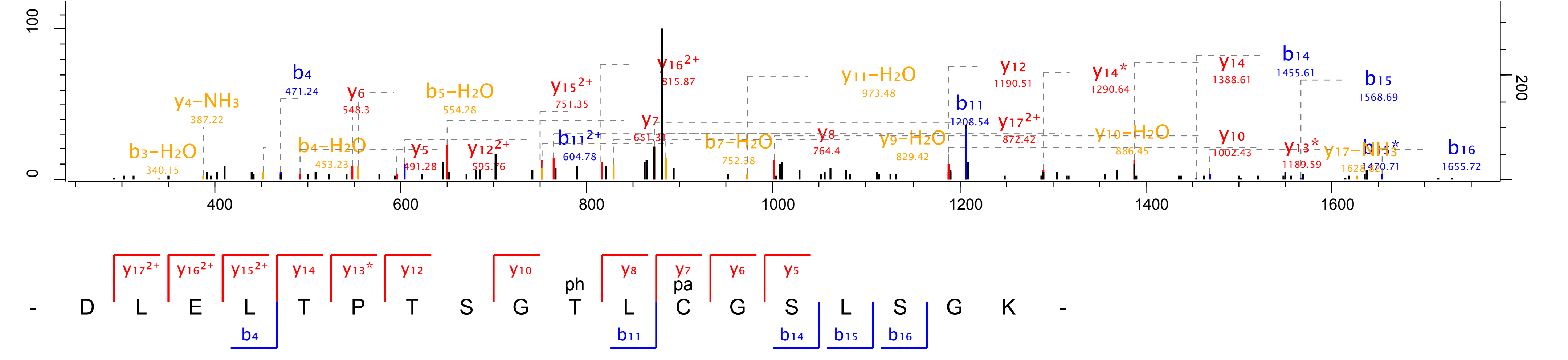

Raw file	Scan	Method	Score	m/z	Gene names
OTNCS_Brainpeppalm_2012Nov30-P1-02	13991	ITMS; CID	203.75	1032.44	Mcoln1

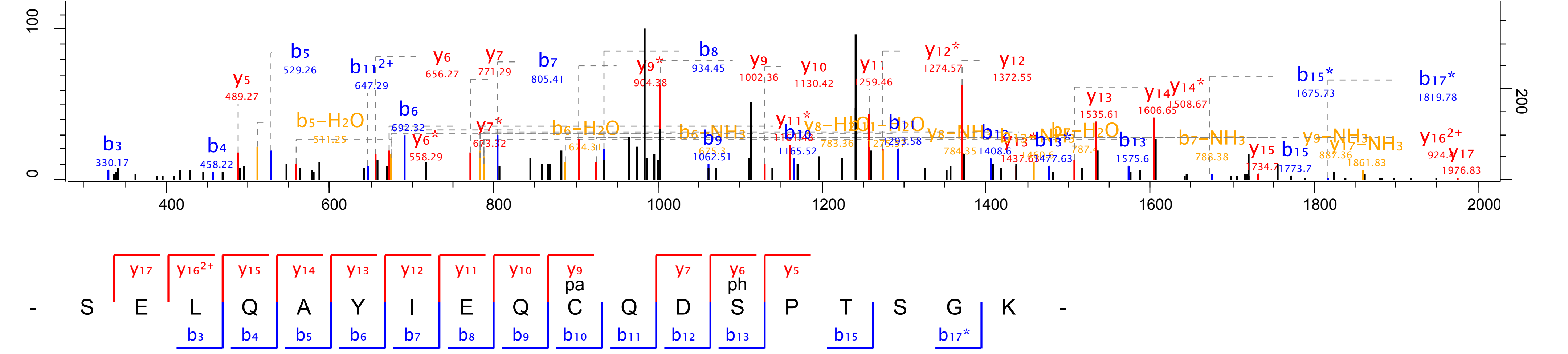

Figure S3. Uncropped image associated with Figure 6e

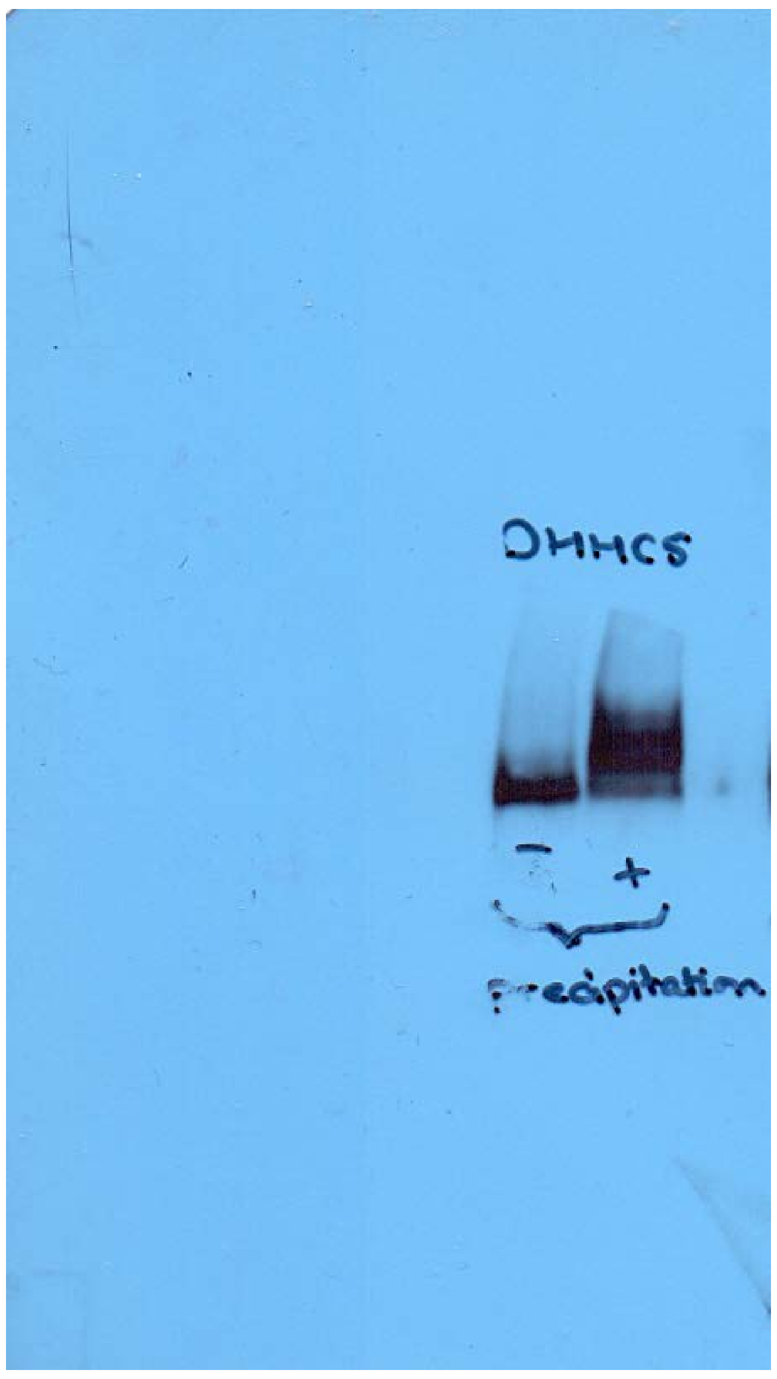

Supplement: Supplementary file 1 — Supplementary Figures [file 41598_2017_4580_MOESM1_ESM.pdf]
